# Associations between parenthood and dementia in men and women: biology or confounding?

**DOI:** 10.1186/s12883-023-03108-7

**Published:** 2023-03-01

**Authors:** Saima Basit, Jan Wohlfahrt, Heather A. Boyd

**Affiliations:** Department of Epidemiology Research, Statens Serum Institut, Artillerivej 5, Copenhagen, Denmark

**Keywords:** Dementia, Alzheimer’s disease, Vascular dementia, Pregnancy, Parity, Epidemiology

## Abstract

**Background:**

High parity and extremes of age at first birth have been linked with increased dementia risk in women, with exposure to pregnancy-associated physiological changes proposed as an explanation. However, confounding by socioeconomic and lifestyle factors could also produce such associations, whereby men would share similar patterns of association. We investigated whether these associations hold for both sexes.

**Methods:**

In a cohort study including all women (*N* = 2,222,638) and men (*N* = 2,141,002) ≥ 40 years of age in 1994–2017 in Denmark, we used Cox regression to evaluate associations between number of children, age at first birth, and dementia risk separately for women and men*.*

**Results:**

During follow-up, 81,413 women and 53,568 men (median age at diagnosis, 83.3 and 80.3 years, respectively) developed dementia. Compared with having one child, having two or more children was associated with modest decreases in overall dementia risk in both sexes (hazard ratio [HR] range 0.82–0.91, P_difference men vs. women_ = 0.07). Although the associations between childlessness and overall dementia risk differed statistically for men and women, the association magnitudes differed only slightly (HR_men_ 1.04, 95% confidence interval [CI] 1.01–1.06; HR_women_ 0.99, 95% CI 0.97–1.01; *P* = 0.002). Associations between age at becoming a parent and overall dementia were also similar for women and men, with the exception of older (≥ 40 years) first-time parents (HR_men_ 1.00, 95% CI 0.96–1.05; HR_women_ 0.92, 95% CI 0.86–0.98; *P *= 0.01). With few exceptions, sub-analyses by dementia subtype and timing of onset also revealed similar patterns and effect magnitudes for women and men.

**Conclusions:**

Associations between number of children, age at becoming a parent, and dementia risk were similar for both sexes. Lifestyle and socioeconomic factors are more likely to explain the observed associations than normal pregnancy-related physiological changes.

**Supplementary Information:**

The online version contains supplementary material available at 10.1186/s12883-023-03108-7.

## Background

Incurable and untreatable, dementia is emerging as a leading cause of morbidity and mortality worldwide, with the economic and social costs of dementia care placing increasing burdens on society [1–4]. Because dementia may affect women disproportionately [5], interest in sex-specific risk factors for dementia has intensified [6, 7]. Since factors that affect brain health early in adulthood may be especially relevant, the potential impact of pregnancy on dementia risk in women is of particular interest.

Pregnancy complications such as preeclampsia and stillbirth are associated with an increased risk of dementia [8–10], suggesting that vascular pathology during pregnancy might be an indicator of later dementia risk. Interestingly, studies have also linked childbirth in general and number of children with Alzheimer’s disease risk in women [11–14], and a recent study found that that having five or more completed pregnancies was associated with a substantial increase in the risk of dementia overall [15]. Repeated exposure to the routine physiological changes associated with pregnancy (including significant changes in endocrine, immunological, and cardiovascular function) has been proposed as an explanation for these observations. Interestingly, however, a new study recently contradicted the previous studies, finding instead a U-shaped relationship between parity and dementia, with similar associations in men and women [16]. The underlying biology of pregnancy has also been suggested to account for the increased risk of Alzheimer’s disease observed in women who are very young (< 20 years) or relatively old (≥ 40 years) when they deliver their first child [17] and the increased risk of dementia overall in women < 25 years of age at first delivery [16].

However, rather than reflecting a causal link between normal pregnancy-associated physiological changes and later dementia, previously observed associations between number of children, age at first birth, and increased dementia risk in women could largely result from uncontrolled confounding by socioeconomic and lifestyle factors. Factors such as education, income, health-seeking behavior, access to healthcare, social engagement in the community, nutrition, stress, smoking, and alcohol use are all associated with reproductive patterns. Uncontrolled confounding by social factors and large between-country differences in the distributions of these factors and in their associations with reproductive patterns might also explain the between-study variation that exists in observed association magnitudes, direction, and patterns.

In a nationwide cohort study of more than 4 million persons, we examined associations between number of children and age at becoming a parent with dementia risk in both women and men. By examining the associations in both sexes under the same study conditions, we could evaluate how likely it was that observed associations could be explained by pregnancy-related factors rather than by uncontrolled confounding unrelated to the biology of pregnancy; if the observed associations were equally strong for men and women, an underlying mechanism based on female sex hormones would be unlikely.

## Methods

### Data sources and cohort

The Civil Registration System registers all Danish residents using unique personal identification numbers and updates information on demographics, kinship, and vital status daily [18]. The National Patient Register contains information on all hospital discharge diagnoses assigned since 1977 and all outpatient diagnoses assigned since 1995, registered using International Classification of Diseases (ICD) codes [19]. The Causes of Death Register holds information on the causes (underlying and contributing) of deaths in Denmark since 1970 [20]. The National Prescription Register contains individual-level information on all prescriptions filled in Denmark since 1994, recorded using Anatomic Therapeutic Chemical (ATC) codes [21].

Using information from the Civil Registration System, we constructed a study cohort consisting of all persons who were ≥ 40 years of age and resident in Denmark at some point between 1994 and 2017. Cohort members were followed from 40 years of age or 1 January 1994 (when ICD-10 codes were introduced in Denmark, allowing more reliable sub-classification of dementia), whichever came later, until the first of dementia, death, emigration, designated “missing” in the Civil Registration System, or 30 May 2017 (end of follow-up). Persons who died or emigrated before 1994 were not eligible to be included in the cohort. Persons with a diagnosis of dementia before the start of follow-up (*N* = 2839) were excluded.

### Exposure: number of children and age at becoming a parent

We obtained information on number of live-born children and age at becoming a parent (also referred to as age at first birth) through the Civil Registration System. Number of children was treated as a time-dependent variable and increased with the birth of additional children during follow-up.

### Outcome: Dementia

Dementia was defined as registration of a dementia code in the National Patient Register during follow-up and was classified as Alzheimer’s disease (ICD-10 codes F00.0-F00.9, G30.0, G30.1, G30.8, G30.9), vascular dementia (ICD-10 codes F01.0-F01.9), and other/unspecified dementia (ICD-10 code F02.0, F03.9). (The ICD-8 codes 290.00, 290.10, 290.11, 290.19, and 299.99 were also used when excluding persons diagnosed with dementia before the start of follow-up). Dementia registered before 65 years of age was considered early-onset dementia; dementia diagnoses first registered ≥ 65 years were considered late-onset dementia.

### Covariates

We adjusted all analyses for birth year (5-year intervals), age (time-dependent variable), and key comorbidities present at the start of follow-up or developing during the study period (time-dependent variables). Comorbidities were identified using the National Patient Register and the Causes of Death Register, based on the following ICD-8 and-10 codes: stroke, 433.xx, 436.xx, I63.0-I63.9; myocardial infarction, 410.xx, I21.0-I23.9; ischemic heart disease, 411.xx-414.xx, 420.xx-429.xx, I20.0-I20.9, I24.0-I25.9; heart failure, 427.09–427.19, 427.99, 428.99, 782.49, I50.0-I50.9; diabetes, 249.00–250.09, E10.0-E14.9; renal disease, 400.39, 403.99, 404.99, I12.0-I13.9, I15.0, I15.1, N18.0-N18.9. Hypertension was identified based on the filling of two prescriptions for anti-hypertensive medication (ATC codes C02-03, C07-09 registered in the National Prescription Register) within a 6-month period.

### Analyses

Using Cox regression with age as the underlying time, we estimated hazard ratios for dementia overall and by subtype, comparing persons with no children and those with two or more children to persons with one child. Among persons with one or more children, we also analyzed the association between age at first birth and dementia. To evaluate whether there were sex-specific differences in the associations of interest, we included parental sex in our models and evaluated the interaction between sex and the reproductive history variables. When analyzing associations with dementia subtypes, we used competing risk methodology [22] to estimate separate hazard ratios for each dementia subtype. In these analyses, follow-up ended with a person’s first dementia diagnosis, regardless of subtype, but only cases of the specific dementia subtype being analyzed counted as an event. (As in the overall analyses, follow-up also ended at death, emigration, registration as “missing”, or the end of the study period). To assess the importance of death as a competing risk in our main analyses, we also evaluated associations between number of children, age at first birth, and all-cause mortality in a Cox regression analysis with all-cause mortality as the outcome (identified using the Civil Registration System) and with censoring at the first dementia diagnosis.

We assessed adherence to the proportional hazards assumption by examining the interaction between the exposure variables and age (< 65 vs ≥ 65 years). All analyses were performed using SAS statistical software, version 9.4 (SAS Institute, Inc., Cary, N.C.).

## Results

We followed 2 222 638 women and 2 141 002 men for 32 656 082 and 30 583 988 person-years, respectively, with a median follow-up of 15.5 years (interquartile range [IQR] 7.7–23.4 years) for women and 14.8 years (IQR 7.3–23.4) for men. During follow-up, 134 981 persons developed dementia, of whom 81 413 (60.3%) were women and 53 568 (39.7%) were men. The initial dementia diagnosis was vascular dementia for 6790 women (8.3%) and 6443 men (12.0%), Alzheimer’s disease for 23 003 women (28.3%) and 13 743 men (25.7%), and other/unspecified for 51 620 women (63.4%) and 33 382 men (62.3%). The median age at diagnosis was 83.3 years (IQR 77.7–87.9 years) for women and 80.3 years (IQR 73.9–85.4 years) for men. Table 1 shows the characteristics of the study cohort at the start of follow-up.

**Table 1:** Characteristics at the start of follow-up^a^ for a cohort of persons ≥ 40 years old followed in the period 1994–2017 in Denmark

Characteristic at the start of follow-up	Women (*n* = 2 222 638)		Men (*n* = 2 141 002)	
	Number^b^	%	Number^b^	%
Number of children				
0	564 573	25.40	592 939	27.69
1	401 421	18.06	373 570	17.45
2	783 069	35.23	726 956	33.95
3	347 885	15.65	325 197	15.19
4	92 119	4.14	89 209	4.17
≥ 5	33 571	1.51	33 131	1.55
Age (years) at birth of first child				
No children	555 211	24.98	561 562	26.23
< 20	179 332	8.07	41 620	1.94
20–24	606 326	27.28	384 139	17.94
25–29	523 463	23.55	582 783	27.22
30–34	245 374	11.04	357 208	16.68
35–39	91 564	4.12	148 242	6.92
≥ 40	21 368	0.96	65 448	3.06
Year of birth				
< 1920	247 881	11.15	146 530	6.84
1920–1929	243 485	10.95	207 143	9.68
1930–1939	267 286	12.03	261 136	12.20
1940–1949	379 109	17.06	394 804	18.44
1950–1959	378 924	17.05	397 864	18.58
1960–1969	416 376	18.73	438 513	20.48
≥ 1970	289 577	13.03	295 012	13.78
Age (years) at the start of follow-up				
< 45	1 121 354	50.45	1 169 290	54.61
45–49	206 725	9.30	216 958	10.13
50–54	163 816	7.37	168 910	7.89
55–59	138 038	6.21	136 313	6.37
60–64	124 592	5.61	117 222	5.48
65–69	122 810	5.53	1081 96	5.05
≥ 70	345 303	15.54	224 113	10.47
Heart disease				
Yes	21 543	0.97	22 857	1.07
No	2 201 095	99.03	2 118 145	98.93
Stroke				
Yes	54 314	2.44	78 281	3.66
No	2 168 324	97.56	2 062 721	96.34
Kidney disease				
Yes	2323	0.10	2976	0.14
No	2 220 315	99.90	2 138 026	99.86
Diabetes				
Yes	35 229	1.59	38 608	1.80
No	2 187 409	98.41	2 102 394	98.20
Hypertension				
Yes	69 887	3.14	42 142	1.97
No	2 152 751	96.86	2 098 860	98.03

^a^Follow-up began on 1 January 1994 or at 40 years of age, whichever came later. Accordingly, persons who began follow-up in 1994 represented a range of ages from 40 to 109 years. In contrast, persons who began follow-up at a later date were necessarily all 40 years of age at study entry, as they had aged into the cohort. This condition accounts for the overrepresentation of persons < 45 years of age at the start of follow-up

^b^Note that these totals reflect the numbers at the start of follow-up. Because relatively young persons were overrepresented at this point, rates of heart disease, stroke, kidney disease, diabetes, and hypertension are lower than might be expected if the age distribution of the cohort at the start of follow-up reflected the age distribution in the general population. Since with the exception of birth year, age at birth of the first child, and age at the start of follow-up, all variables were time-dependent, variable distributions will have changed during the study period

**Fig. 1:**
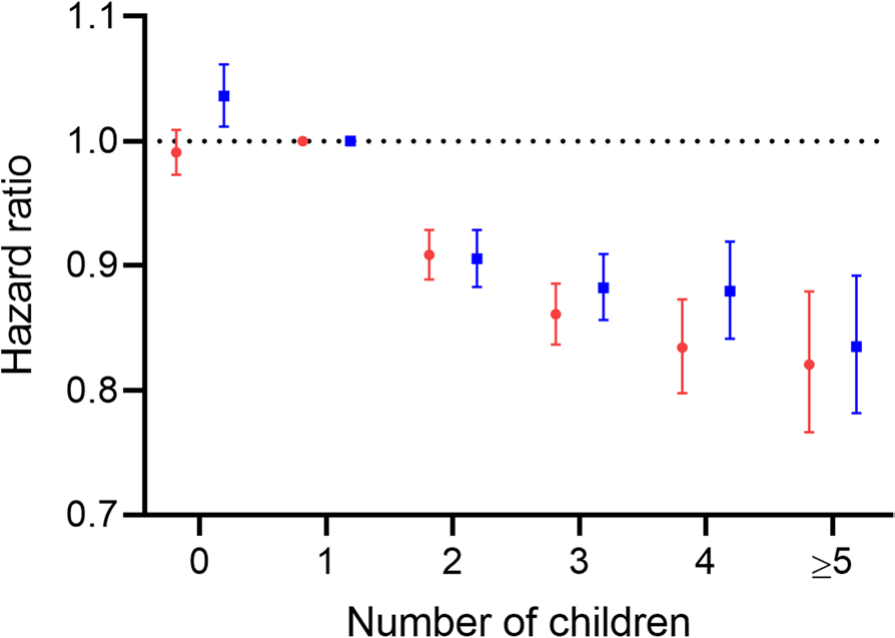
Associations between number of children and overall dementia for men and women, 1994–2017, Denmark. Hazard ratios with 95% confidence intervals compare the risks of dementia among women (red) and men (blue) with different numbers of children. All hazard ratios are adjusted for birth year (5-year intervals), cardiovascular disease, stroke, hypertension, chronic kidney disease and diabetes; age was the underlying time scale in the Cox model

The overall pattern of association between number of children and overall dementia risk differed statistically for men and women (*p* = 0.009, Fig. 1; Supplementary Table 1), largely due to a small but influential difference between childless men and women. Compared with having one child, being childless was associated with a slight increase in overall dementia risk in men (hazard ratio [HR] 1.04, 95% confidence interval [CI] 1.01–1.06) but was not associated with dementia risk in women (HR 0.99, 95% CI 0.97–1.01) (*p* = 0.002 for difference). In contrast, patterns of association among persons with two or more children did not differ for men and women (comparison of the overall pattern of association in men and women with children: *p* = 0.07 for difference). Compared with having only one child, having two, three, four, or five or more children was associated with modest decreases in overall dementia risk in both men and women (hazard ratio [HR] range: 0.82–0.91) (Fig. 1; Supplementary Table 1).

Patterns of association by number of children were generally comparable for men and women for both early- (*p* = 0.24) and late-onset dementia (*p* = 0.20) (Supplementary Figure 1 and Supplementary Table 2). Sex-specific association magnitudes only differed for the association between childlessness and late-onset dementia (HR_men_ 1.01, 95% CI 0.99–1.04; HR_women_ 0.98, 95% CI 0.97–1.00; *p* = 0.04) and even then, the difference in HRs was small.

Similarly, there was little evidence of sex-specific differences in association magnitudes for persons with two or more children for any dementia subtypes (comparison of the overall pattern of association in men and women with children: *p* = 0.30, 0.46, and 0.19 for vascular dementia, Alzheimer’s disease, and other/unspecified dementia, respectively) (Supplementary Figure 2 and Supplementary Table 3). Associations between childlessness and both vascular dementia and Alzheimer’s disease were also of similar magnitude for men and women (vascular dementia: HR_men_ 1.00, 95% CI 0.93–1.07, HR_women_ 0.97, 95% CI 0.91–1.04, *p* = 0.49; Alzheimer’s disease: HR_men_ 0.84, 95% CI 0.79–0.88, HR_women_ 0.89, 95% CI 0.85–0.92, *p* = 0.08). However, childlessness was associated with a greater increase in the risk of other/unspecified dementia in men (HR 1.11, 95% CI 1.08–1.14) than in women (HR 1.03, 95% CI 1.01–1.05) (*p* < 0.0001). Similar patterns were observed when dementia subtype and timing of dementia onset were examined together (Supplementary Figure 3 and Supplementary Table 4).

**Fig. 2:**
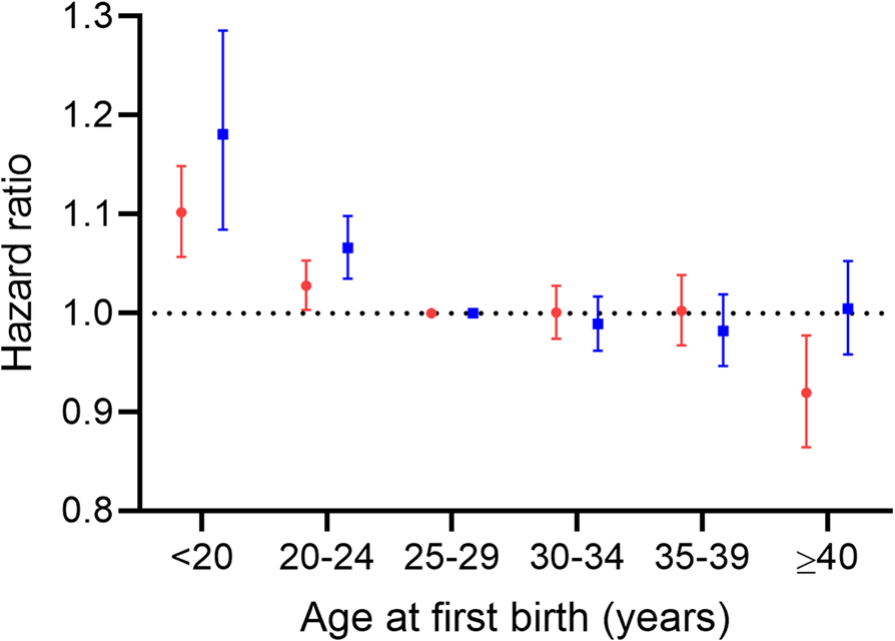
Associations between age at first birth and overall dementia for men and women, 1994–2017, Denmark. Hazard ratios with 95% confidence intervals compare the risks of dementia among women (red) and men (blue) with different ages at first childbirth. All hazard ratios are adjusted for birth year (5-year intervals), cardiovascular disease, stroke, hypertension, chronic kidney disease and diabetes; age was the underlying time scale in the Cox model

The overall pattern of association between age at first birth and overall dementia risk differed statistically for men and women (*p* = 0.02), owing largely to a modest difference in the associations observed for older (≥ 40 years) first-time parents (Fig. 2; Supplementary Table 5); older first-time mothers had a slightly reduced risk of dementia overall, compared with women who were 25–29 years of age at first birth (HR 0.92, 95% CI 0.86–0.98), whereas no reduction in dementia risk was observed for older first-time fathers (HR 1.00, 95% CI 0.96–1.05) (*p* = 0.01 for difference). Becoming a parent at a young age (< 20 years) was associated with a modest increase in overall dementia risk in both sexes (HR_men_ 1.18, 95% CI 1.08–1.29, HR_women_ 1.10, 95% CI 1.06–1.15, *p* = 0.17) (Fig. 2).

The strength of the associations between age at first birth and risk of early-onset dementia differed little for men and women, regardless of age category (*p* = 0.78 for overall difference, Supplementary Figure 4 and Supplementary Table 6). The overall pattern of association between age at first birth and risk of late-onset dementia was also similar for both sexes (*p* = 0.08, Supplementary Figure 4 and Supplementary Table 6), with the exception of a small difference in sex-specific association magnitudes for the oldest (≥ 40 years) first-time parents (HR_men_ 1.01, 95% CI 0.96–1.06, HR_women_ 0.92, 95% CI 0.86–0.98, *p* = 0.01). There was little evidence of sex-specific differences in patterns of association between age at first birth and either vascular dementia (*p* = 0.98) or unspecified dementia (*p* = 0.18) (Supplementary Table 7). Overall, sex-specific patterns of association differed for Alzheimer’s disease (*p* = 0.02), but within age categories, any sex-specific differences were small (Supplementary Figure 5 and Supplementary Table 7). Examining dementia subtype and timing of onset together did not reveal evidence of meaningful differences in the associations between age at first birth and dementia risk in women and men (Supplementary Figure 6 and Supplementary Table 8).

The pattern of associations for mortality were similar to the patterns we observed for dementia, although the associations were somewhat more pronounced for childless persons and persons who were young when they became parents (Supplementary Tables 9 and 10). The mortality results suggest that for these groups (childless persons and persons who became parents at a young age) in particular, the hazard ratios for dementia (the focus in this study) cannot necessarily be used to discuss cumulative risk ratios.

## Discussion

In this population-based cohort study of more than 4 million persons, we found that, with few exceptions, patterns of association between number of children, age at becoming a parent, and dementia risk were the same for men and women; where sex-specific estimates did differ, the absolute differences in effect magnitude were small. The similarity of our findings in both sexes challenges previous suggestions that pregnancy-related hormonal changes or other normal pregnancy-associated physiological changes might explain observed associations between number of children and dementia risk in women.

Rather than being evidence of a causal mechanism involving pregnancy-related hormonal factors, the observed associations between reproductive factors and dementia are more likely due to uncontrolled confounding by social factors that include educational attainment, income, employment, cohabitation status, social network, and lifestyle factors. In early adulthood, these factors may affect reproductive patterns and decisions to have (or not have) children, how many children to have, and when to have them; some are also associated with involuntary childlessness [23–26]. In turn, reproductive patterns and decisions can exert effects on later socioeconomic status, social behaviours, stress levels, and lifestyle. Since demographic, social, lifestyle, and health factors both early and later in life are also associated with later dementia risk [27–31], these factors are obvious potential confounders or mediators. For example, the observation that childlessness was associated with increased risks of early-onset dementia in both sexes might reflect differences in social contact and support experienced by persons with and without family networks [32, 33]; alternatively, persons in families with a history of dementia may choose not to have children so as not to pass on a genetic predisposition to dementia. Similarly, becoming a parent at a young age is associated with lower socioeconomic status [34], which is a known risk factor for dementia [27, 29, 31]. Even where results differed for men and women, confounding is a more plausible explanation than the biology of pregnancy; associations between social factors and reproductive decisions differ for men and women [23, 25, 26].

Although our results linking extremes of age at first birth with modestly increased dementia risks are consistent with the results of previous studies [16, 17], our finding of similar associations for men and women suggests uncontrolled confounding as a more likely explanation for the associations than female reproductive biology. With the exception of findings from a recent study based on data from the UK biobank, [16] our results for number of children differed markedly from those reported by others. While most previous studies reported that motherhood was associated with Alzheimer’s disease risk and that a woman’s risk appeared to increase with increasing numbers of children [13–15], both we found that being childless was associated with modest increases in dementia risk, whereas having two or more children either was not associated with dementia risk or was associated with modest risk reductions. We found no evidence of a trend in dementia risk with increasing numbers of children and certainly no evidence that having large numbers of children was harmful, contrary to the previous studies [14, 15]. Given our claim that associations between reproductive patterns and dementia risk are subject to substantial confounding by social factors, these between-study differences are not surprising. Existing studies come from various different countries, and there is considerable international variation (and temporal variation) in the distributions of potential confounding factors and the relationships these factors have with both reproductive patterns and dementia [25, 26, 35–37]. For example, in Denmark, which throughout the study period had universal education and healthcare, easy and universal access to contraception, and the right to legal abortion, one might expect that a large proportion of higher-order births were planned. Having many children would therefore not necessarily be associated with lower education or socioeconomic status, unplanned pregnancies, and other factors associated both directly and indirectly (e.g. via poor mental health, including depression [38]) with later dementia risk, as might be more likely in countries lacking such social “safety net” features. We contend that adequate adjustment for confounding by social factors would substantially change the observed associations between reproductive patterns and dementia risk in a way specific to the nation, region, population group, and time period under study.

Although our results suggest that pregnancy per se – that is, the normal physiological changes associated with pregnancy – is unlikely to be associated with increased risk of dementia in women once social factors specific to the population under study are taken into consideration, there is no question that some pregnancy complications are associated with an increased risk of dementia. Recent studies have linked both hypertensive disorders of pregnancy and stillbirth with increased risks of dementia, vascular dementia in particular [8–10]. Furthermore, a genetic polymorphism that increases the risk of Alzheimer’s disease may also be associated with gestational diabetes [39]. A history of pregnancy complications may therefore provide a useful indicator of increased future dementia risk, helping to identify groups of potentially at-risk women who might benefit from additional clinical attention.

While we posit that residual confounding, rather than the biology of pregnancy, is largely responsible for observed associations between number of children, age at first birth, and dementia, we were unable to adjust our results for social and lifestyle factors, as only limited information on such factors is available in the Danish registers. For example, information on smoking, alcohol use and body mass index are only available for women in the first trimester of pregnancy. However, a recent study of reproductive history and risk of dementia adjusted for smoking and other lifestyle factors, including age, Townsend index (a measure of material deprivation), ethnicity, smoking, systolic blood pressure, body mass index, diabetes, total cholesterol, and use of antihypertensive medications and lipid-lowering medications [16]. Even after these adjustments, the study found similar associations between number of children and risk of dementia in men and women, indicating that the influence of confounding by lifestyle factors on the association of interest does not differ meaningfully for men and women. Consequently, it is unlikely that differential residual confounding by these factors is hiding sex-specific differences in association that suggest a pregnancy-related link in women.

Registration of dementia diagnoses is probably incomplete, or at least delayed, because general practitioners, who do not report to the National Patient Register, handle a certain proportion of milder cases of dementia [40]. However, a study of dementia diagnoses registered in the National Patient Register found that 88% of persons with a registered dementia diagnosis did in fact have dementia according to their medical records, and registered diagnoses of Alzheimer’s disease, vascular dementia, and other/unspecified dementia agreed with the diagnosis noted in the medical record for 97%, 96%, and 81% of patients, respectively [41]. Since neither diagnosis nor registration of dementia was likely to have depended on number of children or age at first birth, any misclassification of dementia status was likely to have been non-differential.

Our study’s greatest strength was the inclusion of men, which provided insight into the probable relative importance of social and biological factors in explaining the observed associations. Moreover, the use of the entire Danish population as the study cohort minimized selection bias and provided enormous statistical power. The use of registers ensured the absence of recall bias in the ascertainment of both exposure and outcome.

## Conclusions

Associations between number of children, age at becoming a parent, and dementia risk were generally the same for both sexes, regardless of dementia subtype and timing of onset. Our findings argue against the suggestion that the normal physiological changes associated with pregnancy influence dementia risk in women. Even in the few instances where association magnitudes differed for men and women, the observed associations are more likely to be explained by lifestyle and socioeconomic factors associated with reproductive history than by the biology of pregnancy.

## Supplementary Information

**Additional file 1**: **Supplementary Table 1.** Hazard ratios for dementia overall by number of children in a cohort of individuals ≥40 years in the period 1994-2017 in Denmark. **Supplementary Table 2.** Hazard ratios for dementia overall by number of children and timing of dementia onset, in a cohort of individuals ≥40 years in the period 1994-2017 in Denmark. **Supplementary Table 3.** Hazard ratios for dementia subtypes by number of children in a cohort of individuals ≥40 years in the period 1994-2017 in Denmark. **Supplementary Table 4.** Hazard ratios for dementia subtypes by number of children and timing of dementia onset in a cohort of individuals ≥40 years in the period 1994-2017 in Denmark. **Supplementary Table 5.** Hazard ratios for dementia overall by age at first becoming a parent in a cohort of individuals ≥40 years in the period 1994-2017 in Denmark. **Supplementary Table 6.** Hazard ratios for dementia overall by age at first becoming a parent and timing of dementia onset in a cohort of individuals ≥40 years in the period 1994-2017 in Denmark. **Supplementary Table 7.** Hazard ratios for dementia subtypes by age at first becoming a parent in a cohort of individuals ≥40 years in the period 1994-2017 in Denmark. **Supplementary Table 8.** Hazard ratios for dementia subtypes by age at first becoming a parent and timing of onset of dementia in a cohort of individuals ≥40 years in the period 1994-2017 in Denmark. **Supplementary Figure 1.** Associations between number of children and dementia, by timing of dementia onset, in a cohort of individuals ≥40 years old in the period 1994-2017 in Denmark. **Supplementary Figure 2.** Associations between number of children and dementia, by dementia subtype, in a cohort of individuals ≥40 years old in the period 1994-2017 in Denmark. **Supplementary Figure 3.** Associations between number of children and dementia, by dementia subtype and timing of dementia onset, in a cohort of individuals ≥40 years old in the period 1994-2017 in Denmark. **Supplementary Figure 4.** Associations between age at first birth and overall dementia, by timing of dementia onset, in a cohort of individuals ≥40 years old with ≥1 childbirths in the period 1994-2017 in Denmark. **Supplementary Figure 5.** Associations between age at first birth and overall dementia, by dementia subtype, in a cohort of individuals ≥40 years old with ≥1 childbirths in the period 1994-2017 in Denmark. **Supplementary Figure 6.** Associations between age at first birth and overall dementia, by dementia subtype and timing of dementia onset, in a cohort of individuals ≥40 years old with ≥1 childbirths in the period 1994-2017 in Denmark**.**

## Data Availability

The datasets analysed during the current study were drawn from Danish national registers and do not belong to, and may not be shared by, the authors, except in aggregate (as, for example, in a publication). However, interested parties can obtain the data on which the study was based by submitting a research protocol to the Danish Data Protection Agency (Datatilsynet) and then, once Data Protection Agency permission has been received, applying to the Danish Health Data Authority’s Research Service (Forskerservice) at forskerservice@sundhedsdata.dk.

## Abbreviations

HR: Hazard ratio
95% CI: 95% Confidence interval
