# Supplementary material for: Associations between parenthood and dementia in men and women: biology or confounding?
Source: BMC Neurol. 2023 Mar 1;23:90. doi: 10.1186/s12883-023-03108-7 (PMC9976501; doi:10.1186/s12883-023-03108-7)
Supplement: Supplementary file 1 — Additional file 1: Supplementary Table 1. Hazard ratios for dementia overall by number of children in a cohort of individuals ≥40 years in the period 1994-2017 in Denmark. Supplementary Table 2. Hazard ratios for dementia overall by number of children and timing of dementia onset, in a cohort of individuals ≥40 years in the period 1994-2017 in Denmark. Supplementary Table 3. Hazard ratios for dementia subtypes by number of children in a cohort of individuals ≥40 years in the period 1994-2017 in Denmark. Supplementary Table 4. Hazard ratios for dementia subtypes by number of children and timing of dementia onset in a cohort of individuals ≥40 years in the period 1994-2017 in Denmark. Supplementary Table 5. Hazard ratios for dementia overall by age at first becoming a parent in a cohort of individuals ≥40 years in the period 1994-2017 in Denmark. Supplementary Table 6. Hazard ratios for dementia overall by age at first becoming a parent and timing of dementia onset in a cohort of individuals ≥40 years in the period 1994-2017 in Denmark. Supplementary Table 7. Hazard ratios for dementia subtypes by age at first becoming a parent in a cohort of individuals ≥40 years in the period 1994-2017 in Denmark. Supplementary Table 8. Hazard ratios for dementia subtypes by age at first becoming a parent and timing of onset of dementia in a cohort of individuals ≥40 years in the period 1994-2017 in Denmark. Supplementary Figure 1. Associations between number of children and dementia, by timing of dementia onset, in a cohort of individuals ≥40 years old in the period 1994-2017 in Denmark. Supplementary Figure 2. Associations between number of children and dementia, by dementia subtype, in a cohort of individuals ≥40 years old in the period 1994-2017 in Denmark. Supplementary Figure 3. Associations between number of children and dementia, by dementia subtype and timing of dementia onset, in a cohort of individuals ≥40 years old in the period 1994-2017 in Denmark. Supplementary Figure [file 12883_2023_3108_MOESM1_ESM.docx]

**SUPPLEMENTARY MATERIAL**

**Associations between parenthood and dementia in men and women: biology or confounding?**

Saima Basit, Jan Wohlfahrt, Heather A. Boyd

Supplementary Table 1. Hazard ratios for dementia overall by number of children in a cohort of individuals ≥40 years in the period 1994-2017 in Denmark

Supplementary Table 2. Hazard ratios for dementia overall by number of children and timing of dementia onset, in a cohort of individuals ≥40 years in the period 1994-2017 in Denmark

Supplementary Table 3. Hazard ratios for dementia subtypes by number of children in a cohort of individuals ≥40 years in the period 1994-2017 in Denmark

Supplementary Table 4. Hazard ratios for dementia subtypes by number of children and timing of dementia onset in a cohort of individuals ≥40 years in the period 1994-2017 in Denmark

Supplementary Table 5. Hazard ratios for dementia overall by age at first becoming a parent in a cohort of individuals ≥40 years in the period 1994-2017 in Denmark

Supplementary Table 6. Hazard ratios for dementia overall by age at first becoming a parent and timing of dementia onset in a cohort of individuals ≥40 years in the period 1994-2017 in Denmark

Supplementary Table 7. Hazard ratios for dementia subtypes by age at first becoming a parent in a cohort of individuals ≥40 years in the period 1994-2017 in Denmark

Supplementary Table 8. Hazard ratios for dementia subtypes by age at first becoming a parent and timing of onset of dementia in a cohort of individuals ≥40 years in the period 1994-2017 in Denmark

Supplementary Figure 1. Associations between number of children and dementia, by timing of dementia onset, in a cohort of individuals ≥40 years old in the period 1994-2017 in Denmark

Supplementary Figure 2. Associations between number of children and dementia, by dementia subtype, in a cohort of individuals ≥40 years old in the period 1994-2017 in Denmark

Supplementary Figure 3. Associations between number of children and dementia, by dementia subtype and timing of dementia onset, in a cohort of individuals ≥40 years old in the period 1994-2017 in Denmark

Supplementary Figure 4. Associations between age at first birth and overall dementia, by timing of dementia onset, in a cohort of individuals ≥40 years old with ≥1 childbirths in the period 1994-2017 in Denmark

Supplementary Figure 5. Associations between age at first birth and overall dementia, by dementia subtype, in a cohort of individuals ≥40 years old with ≥1 childbirths in the period 1994-2017 in Denmark

Supplementary Figure 6. Associations between age at first birth and overall dementia, by dementia subtype and timing of dementia onset, in a cohort of individuals ≥40 years old with ≥1 childbirths in the period 1994-2017 in Denmark

**Supplementary Table 1. Hazard ratios for dementia overall by number of children in a cohort of individuals ≥40 years in the period 1994-2017 in Denmark**

| Number of children | Men | | | | Women | | | | P-value for difference between men and women |
| --- | --- | --- | --- | --- | --- | --- | --- | --- | --- |
|  | Person-years (x 10^3^) | No. of events | HR | 95% CI | Person-years (x 10^3^) | No. of events | HR | 95% CI |  |
| 0 | 6322 | 16 662 | 1.04 | 1.01-1.06 | 6018 | 35 179 | 0.99 | 0.97-1.01 | 0.002 |
| 1 | 5082 | 11 643 | 1 | Ref | 5968 | 19 756 | 1 | Ref |  |
| 2 | 11 532 | 14 460 | 0.91 | 0.88-0.93 | 12 771 | 16 206 | 0.91 | 0.89-0.93 | 0.11 |
| 3 | 5418 | 7379 | 0.88 | 0.86-0.91 | 5766 | 7204 | 0.86 | 0.84-0.89 | 0.09 |
| 4 | 1598 | 2452 | 0.88 | 0.84-0.92 | 1568 | 2204 | 0.83 | 0.80-0.87 | 0.09 |
| ≥5 | 632 | 972 | 0.84 | 0.78-0.89 | 566 | 864 | 0.82 | 0.77-0.88 | 0.52 |

CI, confidence interval. HR, hazard ratio.

All hazard ratios are adjusted for birth year (5-year intervals) cardiovascular disease, stroke, hypertension, chronic kidney disease and diabetes; age was the underlying time scale in the Cox model.

**Supplementary Table 2. Hazard ratios for dementia overall by number of children and timing of dementia onset, in a cohort of individuals ≥40 years in the period 1994-2017 in Denmark**

| Number of children | Men | | | | Women | | | | P-value for difference between men and women |
| --- | --- | --- | --- | --- | --- | --- | --- | --- | --- |
|  | Person-years (x 10^3^) | No. of events | HR | 95% CI | Person-years (x 10^3^) | No. of events | HR | 95% CI |  |
| Early-onset dementia | |  |  |  |  |  |  |  |  |
| 0 | 4336 | 1268 | 1.40 | 1.28-1.52 | 2686 | 616 | 1.37 | 1.23-1.53 | 0.82 |
| 1 | 3448 | 798 | 1 | Ref | 3534 | 650 | 1 | Ref |  |
| 2 | 8585 | 1595 | 0.79 | 0.72-0.86 | 9512 | 1315 | 0.76 | 0.70-0.84 | 0.60 |
| 3 | 3859 | 733 | 0.77 | 0.70-0.85 | 4136 | 632 | 0.80 | 0.72-0.89 | 0.69 |
| ≥4 | 1514 | 350 | 0.86 | 0.76-0.97 | 1439 | 211 | 0.70 | 0.60-0.82 | 0.02 |
| Late-onset dementia | |  |  |  |  |  |  |  |  |
| 0 | 1986 | 15 394 | 1.01 | 0.99-1.04 | 3332 | 34 563 | 0.98 | 0.97-1.00 | 0.04 |
| 1 | 1634 | 10 845 | 1 | Ref | 2434 | 19 106 | 1 | Ref |  |
| 2 | 2948 | 12 865 | 0.93 | 0.90-0.95 | 3259 | 14 891 | 0.92 | 0.90-0.94 | 0.43 |
| 3 | 1559 | 6646 | 0.90 | 0.88-0.93 | 1629 | 6572 | 0.87 | 0.84-0.89 | 0.04 |
| ≥4 | 715 | 3074 | 0.87 | 0.84-0.91 | 695 | 2857 | 0.84 | 0.81-0.88 | 0.23 |

CI, confidence interval. HR, hazard ratio.

All hazard ratios are adjusted for birth year (5-year intervals), cardiovascular disease, stroke, hypertension, chronic kidney disease and diabetes; age was the underlying time scale in the Cox model.

**Supplementary Table 3. Hazard ratios for dementia subtypes by number of children in a cohort of individuals ≥40 years in the period 1994-2017 in Denmark**

| Number of children | Men | | | | Women | | | | P-value for difference between men and women |
| --- | --- | --- | --- | --- | --- | --- | --- | --- | --- |
|  | Person-years (x 10^3^) | No. of events | HR | 95% CI | Person-years (x 10^3^) | No. of events | HR | 95% CI |  |
| Vascular dementia | |  |  |  |  |  |  |  |  |
| 0 | 6322 | 1693 | 1.00 | 0.93-1.07 | 6018 | 2650 | 0.97 | 0.91-1.04 | 0.49 |
| 1 | 5082 | 1338 | 1 | Ref | 5968 | 1706 | 1 | Ref |  |
| 2 | 11 532 | 1895 | 0.95 | 0.89-1.03 | 12 771 | 1444 | 0.91 | 0.84-0.97 | 0.09 |
| 3 | 5418 | 1001 | 0.94 | 0.87-1.03 | 5766 | 679 | 0.89 | 0.81-0.97 | 0.64 |
| ≥4 | 2230 | 516 | 1.02 | 0.92-1.13 | 2134 | 311 | 0.89 | 0.79-1.01 | 0.27 |
| Alzheimer’s disease | |  |  |  |  |  |  |  |  |
| 0 | 6322 | 2767 | 0.84 | 0.79-0.88 | 6018 | 6683 | 0.89 | 0.85-0.92 | 0.08 |
| 1 | 5082 | 2785 | 1 | Ref | 5968 | 5644 | 1 | Ref |  |
| 2 | 11 532 | 4732 | 1.03 | 0.98-1.08 | 12 771 | 6480 | 1.00 | 0.96-1.04 | 0.49 |
| 3 | 5418 | 2440 | 0.98 | 0.93-1.04 | 5766 | 2967 | 0.93 | 0.89-0.98 | 0.11 |
| ≥4 | 2230 | 1019 | 0.88 | 0.81-0.94 | 2134 | 1229 | 0.88 | 0.83-0.94 | 0.82 |
| Other/unspecified dementia | | |  |  |  |  |  |  |  |
| 0 | 6322 | 12 202 | 1.11 | 1.08-1.14 | 6018 | 25 846 | 1.03 | 1.01-1.05 | <0.0001 |
| 1 | 5082 | 7520 | 1 | Ref | 5968 | 12 406 | 1 | Ref |  |
| 2 | 11 532 | 7833 | 0.83 | 0.81-0.86 | 12 771 | 8282 | 0.85 | 0.82-0.87 | 0.88 |
| 3 | 5418 | 3938 | 0.82 | 0.79-0.85 | 5766 | 3558 | 0.81 | 0.77-0.84 | 0.18 |
| ≥4 | 2230 | 1889 | 0.83 | 0.79-0.87 | 2134 | 1528 | 0.79 | 0.74-0.83 | 0.05 |

CI, confidence interval. HR, hazard ratio.

All hazard ratios are adjusted for birth year (5-year intervals), cardiovascular disease, stroke, hypertension, chronic kidney disease and diabetes; age was the underlying time scale in the Cox model

**Supplementary Table 4. Hazard ratios for dementia subtypes by number of children and timing of dementia onset in a cohort of individuals ≥40 years in the period 1994-2017 in Denmark**

| Number of children | Men | | | | Women | | | |
| --- | --- | --- | --- | --- | --- | --- | --- | --- |
|  | Person-years (x 10^3^) | No. of events | HR | 95% CI | Person-years (x 10^3^) | No. of events | HR | 95% CI |
| EARLY-ONSET DEMENTIA | | |  |  |  |  |  |  |
| Vascular dementia | | |  |  |  |  |  |  |
| 0 | 4336 | 160 | 1.24 | 0.98-1.58 | 2686 | 56 | 1.40 | 0.97-2.02 |
| 1 | 3448 | 113 | 1 | Ref | 3534 | 59 | 1 | Ref |
| 2 | 8585 | 224 | 0.81 | 0.65-1.02 | 9512 | 112 | 0.74 | 0.54-1.02 |
| 3 | 3859 | 123 | 0.92 | 0.71-1.18 | 4136 | 44 | 0.60 | 0.41-0.89 |
| ≥4 | 1514 | 54 | 0.90 | 0.65-1.25 | 1439 | 21 | 0.69 | 0.42-1.14 |
| Alzheimer’s disease | | |  |  |  |  |  |  |
| 0 | 4336 | 254 | 1.82 | 1.46-2.25 | 2686 | 232 | 1.88 | 1.55-2.29 |
| 1 | 3448 | 124 | 1 | Ref | 3534 | 178 | 1 | Ref |
| 2 | 8585 | 380 | 1.18 | 0.96-1.45 | 9512 | 440 | 0.92 | 0.77-1.09 |
| 3 | 3859 | 154 | 1.04 | 0.82-1.32 | 4136 | 199 | 0.93 | 0.76-1.14 |
| ≥4 | 1514 | 58 | 0.94 | 0.69-1.28 | 1439 | 51 | 0.67 | 0.49-0.91 |
| Other/unspecified dementia | | |  |  |  |  |  |  |
| 0 | 4336 | 854 | 1.33 | 1.19-1.48 | 2686 | 328 | 1.14 | 0.99-1.32 |
| 1 | 3448 | 561 | 1 | Ref | 3534 | 413 | 1 | Ref |
| 2 | 8585 | 991 | 0.70 | 0.63-0.77 | 9512 | 763 | 0.70 | 0.62-0.79 |
| 3 | 3859 | 456 | 0.68 | 0.60-0.77 | 4136 | 389 | 0.77 | 0.67-0.88 |
| ≥4 | 1514 | 238 | 0.83 | 0.71-0.96 | 1439 | 139 | 0.71 | 0.59-0.86 |
| LATE-ONSET DEMENTIA | | |  |  |  |  |  |  |
| Vascular dementia | | |  |  |  |  |  |  |
| 0 | 1986 | 1533 | 0.97 | 0.90-1.05 | 3332 | 2594 | 0.97 | 0.91-1.03 |
| 1 | 1634 | 1225 | 1 | Ref | 2434 | 1647 | 1 | Ref |
| 2 | 2948 | 1671 | 0.98 | 0.91-1.06 | 3259 | 1332 | 0.92 | 0.85-0.99 |
| 3 | 1559 | 878 | 0.95 | 0.87-1.04 | 1629 | 635 | 0.92 | 0.83-1.01 |
| ≥4 | 715 | 462 | 1.04 | 0.93-1.15 | 695 | 290 | 0.91 | 0.80-1.04 |
| Alzheimer’s disease | | |  |  |  |  |  |  |
| 0 | 1986 | 2513 | 0.79 | 0.75-0.83 | 3332 | 6451 | 0.86 | 0.83-0.90 |
| 1 | 1634 | 2661 | 1 | Ref | 2434 | 5466 | 1 | Ref |
| 2 | 2948 | 4352 | 1.03 | 0.98-1.08 | 3259 | 6040 | 1.02 | 0.98-1.05 |
| 3 | 1559 | 2286 | 0.99 | 0.93-1.05 | 1629 | 2768 | 0.94 | 0.90-0.99 |
| ≥4 | 715 | 961 | 0.88 | 0.82-0.95 | 695 | 1178 | 0.91 | 0.85-0.96 |
| Other/unspecified dementia | | |  |  |  |  |  |  |
| 0 | 1986 | 11 348 | 1.09 | 1.06-1.13 | 3332 | 25 518 | 1.03 | 1.01-1.05 |
| 1 | 1634 | 6959 | 1 | Ref | 2434 | 11 993 | 1 | Ref |
| 2 | 2948 | 6842 | 0.86 | 0.83-0.89 | 3259 | 7519 | 0.86 | 0.84-0.89 |
| 3 | 1559 | 3482 | 0.84 | 0.81-0.88 | 1629 | 3169 | 0.81 | 0.77-0.84 |
| ≥4 | 715 | 1651 | 0.83 | 0.79-0.88 | 695 | 1389 | 0.79 | 0.75-0.84 |

CI, confidence interval. HR, hazard ratio.

All hazard ratios are adjusted for birth year (5-year intervals), cardiovascular disease, stroke, hypertension, chronic kidney disease and diabetes; age was the underlying time scale in the Cox model

**Supplementary Table 5. Hazard ratios for dementia overall by age at first becoming a parent in a cohort of individuals ≥40 years in the period 1994-2017 in Denmark**

| Age at first birth (years) | Men | | | | Women | | | | P-value for difference between men and women |
| --- | --- | --- | --- | --- | --- | --- | --- | --- | --- |
|  | Person-years (x 10^3^) | No. of events | HR | 95% CI | Person-years (x 10^3^) | No. of events | HR | 95% CI |  |
| <20 | 737 | 568 | 1.18 | 1.08-1.29 | 3300 | 2925 | 1.10 | 1.06-1.15 | 0.17 |
| 20-24 | 6702 | 7232 | 1.07 | 1.03-1.10 | 10 709 | 13 558 | 1.03 | 1.00-1.05 | 0.05 |
| 25-29 | 9242 | 13 152 | 1 | Ref | 7996 | 14 865 | 1 | Ref |  |
| 30-35 | 4921 | 9165 | 0.99 | 0.96-1.02 | 3259 | 9295 | 1.00 | 0.97-1.03 | 0.37 |
| 35-39 | 1904 | 4338 | 0.98 | 0.95-1.02 | 1139 | 4403 | 1.00 | 0.97-1.04 | 0.52 |
| ≥40 | 852 | 2454 | 1.00 | 0.96-1.05 | 251 | 1188 | 0.92 | 0.86-0.98 | 0.01 |

CI, confidence interval. HR, hazard ratio.

All hazard ratios are adjusted for birth year (5-year intervals), cardiovascular disease, stroke, hypertension, chronic kidney disease and diabetes; age was the underlying time scale in the Cox model

**Supplementary Table 6. Hazard ratios for dementia overall by age at first becoming a parent and timing of dementia onset in a cohort of individuals ≥40 years in the period 1994-2017 in Denmark**

| Age at first birth (years) | Men | | | | Women | | | | P-value for difference between men and women |
| --- | --- | --- | --- | --- | --- | --- | --- | --- | --- |
|  | Person-years (x 10^3^) | No. of events | HR | 95% CI | Person-years (x 10^3^) | No. of events | HR | 95% CI |  |
| Early-onset dementia | |  |  |  |  |  |  |  |  |
| <20 | 597 | 182 | 1.29 | 1.10-1.51 | 2557 | 546 | 1.25 | 1.12-1.40 | 0.75 |
| 20-24 | 4988 | 1287 | 1.14 | 1.05-1.23 | 7688 | 1296 | 1.09 | 0.99-1.20 | 0.54 |
| 25-29 | 6552 | 1255 | 1 | Ref | 5483 | 676 | 1 | Ref |  |
| 30-35 | 3465 | 486 | 0.91 | 0.82-1.01 | 2097 | 214 | 1.00 | 0.86-1.17 | 0.26 |
| 35-39 | 1326 | 183 | 0.96 | 0.82-1.12 | 676 | 65 | 1.02 | 0.79-1.31 | 0.65 |
| ≥40 | 573 | 86 | 1.00 | 0.80-1.24 | 136 | 11 | 0.92 | 0.50-1.66 | 0.84 |
| Late-onset dementia | |  |  |  |  |  |  |  |  |
| <20 | 140 | 386 | 1.14 | 1.02-1.26 | 951 | 2379 | 1.08 | 1.03-1.13 | 0.37 |
| 20-24 | 1714 | 5945 | 1.05 | 1.02-1.09 | 4995 | 12 262 | 1.02 | 1.00-1.05 | 0.13 |
| 25-29 | 2690 | 11 897 | 1 | Ref | 5315 | 14 189 | 1 | Ref |  |
| 30-35 | 1455 | 8679 | 1.00 | 0.97-1.02 | 2862 | 9081 | 1.00 | 0.97-1.03 | 0.57 |
| 35-39 | 578 | 4155 | 0.98 | 0.95-1.02 | 1109 | 4338 | 1.00 | 0.97-1.04 | 0.57 |
| ≥40 | 279 | 2368 | 1.01 | 0.96-1.06 | 220 | 1177 | 0.92 | 0.86-0.98 | 0.01 |

CI, confidence interval. HR, hazard ratio.

All hazard ratios are adjusted for birth year (5-year intervals), cardiovascular disease, stroke, hypertension, chronic kidney disease and diabetes; age was the underlying time scale in the Cox model

**Supplementary Table 7. Hazard ratios for dementia subtypes by age at first becoming a parent in a cohort of individuals ≥40 years in the period 1994-2017 in Denmark**

| Age at first birth (years) | Men | | | | Women | | | | P-value for difference between men and women |
| --- | --- | --- | --- | --- | --- | --- | --- | --- | --- |
|  | Person-years (x 10^3^) | No. of events | HR | 95% CI | Person-years (x 10^3^) | No. of events | HR | 95% CI |  |
| Vascular dementia | |  |  |  |  |  |  |  |  |
| <20 | 737 | 95 | 1.28 | 1.04-1.58 | 3300 | 296 | 1.17 | 1.03-1.34 | 0.53 |
| 20-24 | 6702 | 1011 | 1.02 | 0.94-1.10 | 10 709 | 1247 | 1.02 | 0.94-1.11 | 0.90 |
| 25-29 | 9242 | 1781 | 1 | Ref | 7996 | 1341 | 1 | Ref |  |
| 30-34 | 4921 | 1107 | 0.95 | 0.88-1.03 | 3259 | 802 | 0.98 | 0.89-1.07 | 0.73 |
| 35-39 | 1904 | 499 | 0.97 | 0.87-1.08 | 1139 | 357 | 0.98 | 0.86-1.11 | 0.93 |
| ≥40 | 852 | 258 | 0.99 | 0.86-1.13 | 251 | 97 | 0.98 | 0.80-1.22 | 0.91 |
| Alzheimer’s disease | |  |  |  |  |  |  |  |  |
| <20 | 737 | 138 | 0.91 | 0.77-1.08 | 3300 | 1079 | 1.00 | 0.94-1.07 | 0.26 |
| 20-24 | 6702 | 2346 | 1.04 | 0.98-1.09 | 10 709 | 5422 | 1.01 | 0.97-1.05 | 0.38 |
| 25-29 | 9242 | 4336 | 1 | Ref | 7996 | 5529 | 1 | Ref |  |
| 30-34 | 4921 | 2654 | 0.98 | 0.94-1.03 | 3259 | 2928 | 1.01 | 0.97-1.06 | 0.20 |
| 35-39 | 1904 | 1004 | 0.94 | 0.87-1.01 | 1139 | 1136 | 1.03 | 0.96-1.10 | 0.08 |
| ≥40 | 852 | 498 | 0.96 | 0.87-1.06 | 251 | 226 | 0.84 | 0.73-0.96 | 0.13 |
| Other/unspecified dementia | | |  |  |  |  |  |  |  |
| <20 | 737 | 335 | 1.30 | 1.17-1.46 | 3300 | 1550 | 1.19 | 1.13-1.27 | 0.12 |
| 20-24 | 6702 | 3875 | 1.09 | 1.05-1.14 | 10 709 | 6889 | 1.05 | 1.01-1.08 | 0.10 |
| 25-29 | 9242 | 7035 | 1 | Ref | 7996 | 7995 | 1 | Ref |  |
| 30-34 | 4921 | 5404 | 1.01 | 0.97-1.04 | 3259 | 5565 | 1.00 | 0.96-1.04 | 0.92 |
| 35-39 | 1904 | 2835 | 1.01 | 0.96-1.06 | 1139 | 2910 | 1.00 | 0.95-1.04 | 0.66 |
| ≥40 | 852 | 1698 | 1.04 | 0.98-1.10 | 251 | 865 | 0.94 | 0.88-1.01 | 0.04 |

CI, confidence interval. HR, hazard ratio.

All hazard ratios are adjusted for birth year (5-year intervals), cardiovascular disease, stroke, hypertension, chronic kidney disease and diabetes; age was the underlying time scale in the Cox model

**Supplementary Table 8. Hazard ratios for dementia subtypes by age at first becoming a parent and timing of onset of dementia in a cohort of individuals ≥40 years in the period 1994-2017 in Denmark**

| Age at first birth (years) | Men | | | | Women | | | |
| --- | --- | --- | --- | --- | --- | --- | --- | --- |
|  | Person-years (x 10^3^) | No. of events | HR | 95% CI | Person-years (x 10^3^) | No. of events | HR | 95% CI |
| EARLY-ONSET DEMENTIA | | |  |  |  |  |  |  |
| Vascular dementia | | |  |  |  |  |  |  |
| <20 | 597 | 38 | 1.51 | 1.07-2.14 | 2557 | 60 | 1.71 | 1.16-2.50 |
| 20-24 | 4988 | 178 | 0.91 | 0.75-1.20 | 7688 | 113 | 1.24 | 0.88-1.74 |
| 25-29 | 6552 | 199 | 1 | Ref | 5483 | 48 | 1 | Ref |
| 30-34 | 3465 | 68 | 0.85 | 0.64-1.12 | 2097 | 14 | 0.97 | 0.53-1.75 |
| ≥35* | 1899 | 32 | 0.82 | 0.56-1.19 | 812 | <5 | 0.21 | 0.03-1.50 |
| Alzheimer’s disease | | |  |  |  |  |  |  |
| <20 | 597 | 20 | 0.69 | 0.44-1.08 | 2557 | 128 | 0.98 | 0.79-1.23 |
| 20-24 | 4988 | 238 | 0.99 | 0.83-1.18 | 7688 | 427 | 1.16 | 0.98-1.37 |
| 25-29 | 6552 | 273 | 1 | Ref | 5483 | 214 | 1 | Ref |
| 30-34 | 3465 | 107 | 0.91 | 0.73-1.14 | 2097 | 66 | 0.98 | 0.74-1.29 |
| 35-39 | 1326 | 52 | 1.24 | 0.92-1.67 | 676 | 27 | 1.32 | 0.88-1.97 |
| ≥40 | 573 | 26 | 1.32 | 0.89-1.98 | 136 | 6 | 1.53 | 0.68-3.46 |
| Other/unspecified dementia | | |  |  |  |  |  |  |
| <20 | 597 | 124 | 1.39 | 1.15-1.69 | 2,557 | 358 | 1.37 | 1.19-1.58 |
| 20-24 | 4988 | 871 | 1.22 | 1.11-1.35 | 7688 | 756 | 1.05 | 0.93-1.19 |
| 25-29 | 6552 | 783 | 1 | Ref | 5483 | 414 | 1 | Ref |
| 30-34 | 3465 | 311 | 0.93 | 0.82-1.07 | 2097 | 134 | 1.01 | 0.83-1.23 |
| 35-39 | 1326 | 111 | 0.94 | 0.77-1.15 | 676 | 38 | 0.95 | 0.68-1.33 |
| ≥40 | 573 | 48 | 0.89 | 0.67-1.20 | 136 | <5 | 0.53 | 0.20-1.42 |
| LATE-ONSET DEMENTIA | | |  |  |  |  |  |  |
| Vascular dementia | | |  |  |  |  |  |  |
| <20 | 140 | 57 | 1.14 | 0.87-1.48 | 743 | 236 | 1.11 | 0.96-1.28 |
| 20-24 | 1714 | 833 | 1.03 | 0.95-1.13 | 3022 | 1134 | 1.00 | 0.93-1.09 |
| 25-29 | 2690 | 1582 | 1 | Ref | 2513 | 1293 | 1 | Ref |
| 30-34 | 1455 | 1039 | 0.96 | 0.89-1.04 | 1162 | 788 | 0.97 | 0.89-1.07 |
| ≥35* | 857 | 725 | 0.99 | 0.90-1.09 | 462 | 453 | 0.99 | 0.88-1.11 |
| Alzheimer’s disease | | |  |  |  |  |  |  |
| <20 | 140 | 118 | 0.97 | 0.80-1.16 | 743 | 951 | 1.01 | 0.94-1.09 |
| 20-24 | 1714 | 2108 | 1.04 | 0.99-1.10 | 3022 | 4995 | 1.00 | 0.96-1.05 |
| 25-29 | 2690 | 4063 | 1 | Ref | 2513 | 5315 | 1 | Ref |
| 30-34 | 1455 | 2547 | 0.98 | 0.94-1.04 | 1162 | 2862 | 1.01 | 0.97-1.06 |
| 35-39 | 578 | 952 | 0.92 | 0.86-0.99 | 462 | 1109 | 1.02 | 0.95-1.09 |
| ≥40 | 279 | 472 | 0.95 | 0.85-1.05 | 114 | 220 | 0.82 | 0.72-0.95 |
| Other/unspecified dementia | | |  |  |  |  |  |  |
| <20 | 140 | 211 | 1.26 | 1.10-1.45 | 743 | 1192 | 1.16 | 1.09-1.23 |
| 20-24 | 1714 | 3004 | 1.06 | 1.02-1.11 | 3022 | 6133 | 1.05 | 1.01-1.08 |
| 25-29 | 2690 | 6252 | 1 | Ref | 2513 | 7581 | 1 | Ref |
| 30-34 | 1455 | 5093 | 1.01 | 0.97-1.05 | 1162 | 5431 | 1.00 | 0.96-1.04 |
| 35-39 | 578 | 2724 | 1.01 | 0.97-1.06 | 462 | 2872 | 1.00 | 0.95-1.05 |
| ≥40 | 279 | 1650 | 1.04 | 0.98-1.11 | 114 | 861 | 0.95 | 0.88-1.02 |

CI, confidence interval. HR, hazard ratio.

All hazard ratios are adjusted for birth year (5-year intervals), cardiovascular disease, stroke, hypertension, chronic kidney disease and diabetes; age was the underlying time scale in the Cox model.

* For vascular dementia, the age groups 35-39 and ≥40 were combined due to small numbers of events in each group.

**Supplementary Table 9. Hazard ratios for all_cause mortality by number of children in a cohort of individuals ≥40 years in the period 1994-2017 in Denmark**

| Number of children | Men | | | | Women | | | | P-value for difference between men and women |
| --- | --- | --- | --- | --- | --- | --- | --- | --- | --- |
|  | Person-years (x 10^3^) | No. of events | HR | 95% CI | Person-years (x 10^3^) | No. of events | HR | 95% CI |  |
| 0 | 6322 | 209 382 | 1.25 | 1.24-1.26 | 6018 | 271 465 | 1.13 | 1.12-1.14 | <.0001 |
| 1 | 5082 | 122 433 | 1 | Ref | 5968 | 129 781 | 1 | Ref |  |
| 2 | 11 532 | 148 869 | 0.80 | 0.80-0.81 | 12 771 | 112 910 | 0.82 | 0.82-0.83 | 0.04 |
| 3 | 5418 | 74 674 | 0.79 | 0.78-0.79 | 5766 | 51 964 | 0.80 | 0.79-0.81 | 0.69 |
| 4 | 1598 | 25 944 | 0.83 | 0.82-0.85 | 1568 | 17 005 | 0.84 | 0.83-0.85 | 0.18 |
| ≥5 | 632 | 12 186 | 0.92 | 0.90-0.93 | 566 | 7904 | 0.97 | 0.95-1.00 | 0.01 |

CI, confidence interval. HR, hazard ratio.

All hazard ratios are adjusted for birth year (5-year intervals) cardiovascular disease, stroke, hypertension, chronic kidney disease and diabetes; age was the underlying time scale in the Cox model.

**Supplementary Table 10. Hazard ratios for all_cause mortality overall by age at first becoming a parent in a cohort of individuals ≥40 years in the period 1994-2017 in Denmark**

| Age at first birth (years) | Men | | | | Women | | | | P-value for difference between men and women |
| --- | --- | --- | --- | --- | --- | --- | --- | --- | --- |
|  | Person-years (x 10^3^) | No. of events | HR | 95% CI | Person-years (x 10^3^) | No. of events | HR | 95% CI |  |
| <20 | 737 | 568 | 1.36 | 1.34-1.39 | 3300 | 2925 | 1.35 | 1.33-1.37 | <.0001 |
| 20-24 | 6702 | 7232 | 1.14 | 1.13-1.15 | 10 709 | 13 558 | 1.09 | 1.08-1.10 | <.0001 |
| 25-29 | 9242 | 13 152 | 1 | Ref | 7996 | 14 865 | 1 | Ref |  |
| 30-35 | 4921 | 9165 | 0.98 | 0.97-0.98 | 3259 | 9295 | 1.02 | 1.01-1.03 | <.0001 |
| 35-39 | 1904 | 4338 | 0.98 | 0.97-0.99 | 1139 | 4403 | 1.02 | 1.01-1.04 | <.0001 |
| ≥40 | 852 | 2454 | 1.01 | 0.99-1.02 | 251 | 1188 | 1.02 | 1.00-1.04 | <.0001 |

CI, confidence interval. HR, hazard ratio.

All hazard ratios are adjusted for birth year (5-year intervals), cardiovascular disease, stroke, hypertension, chronic kidney disease and diabetes; age was the underlying time scale in the Cox model

**Supplementary Figure 1. Associations between number of children and dementia, by timing of dementia onset, in a cohort of individuals ≥40 years old in the period 1994-2017 in Denmark.** Hazard ratios with 95% confidence intervals compare the risks of a) early-onset dementia (onset <65 years) and b) late-onset dementia (onset ≥65 years) among women (red) and men (blue) with different numbers of children. All hazard ratios are adjusted for birth year (5-year intervals), cardiovascular disease, stroke, hypertension, chronic kidney disease and diabetes; age was the underlying time scale in the Cox model.


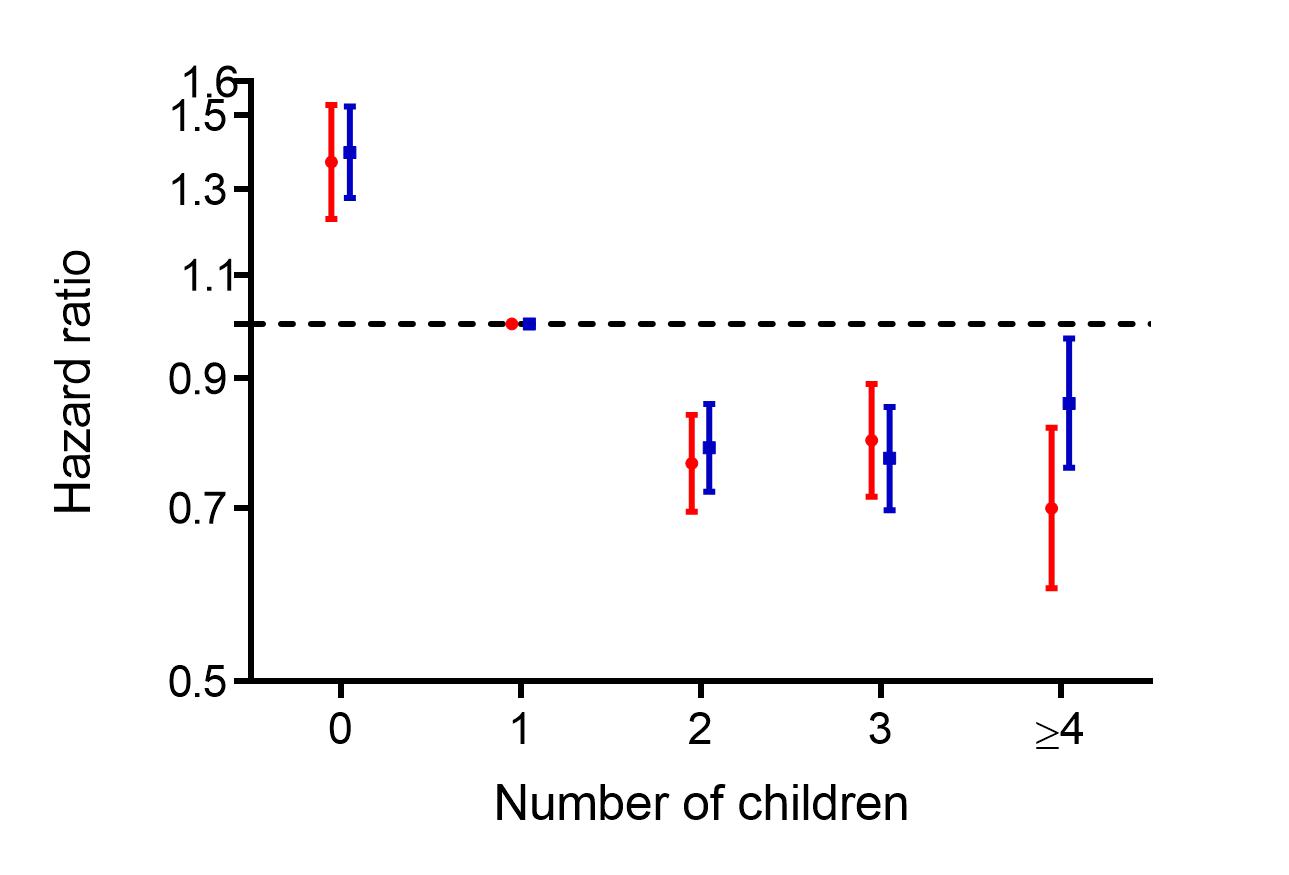

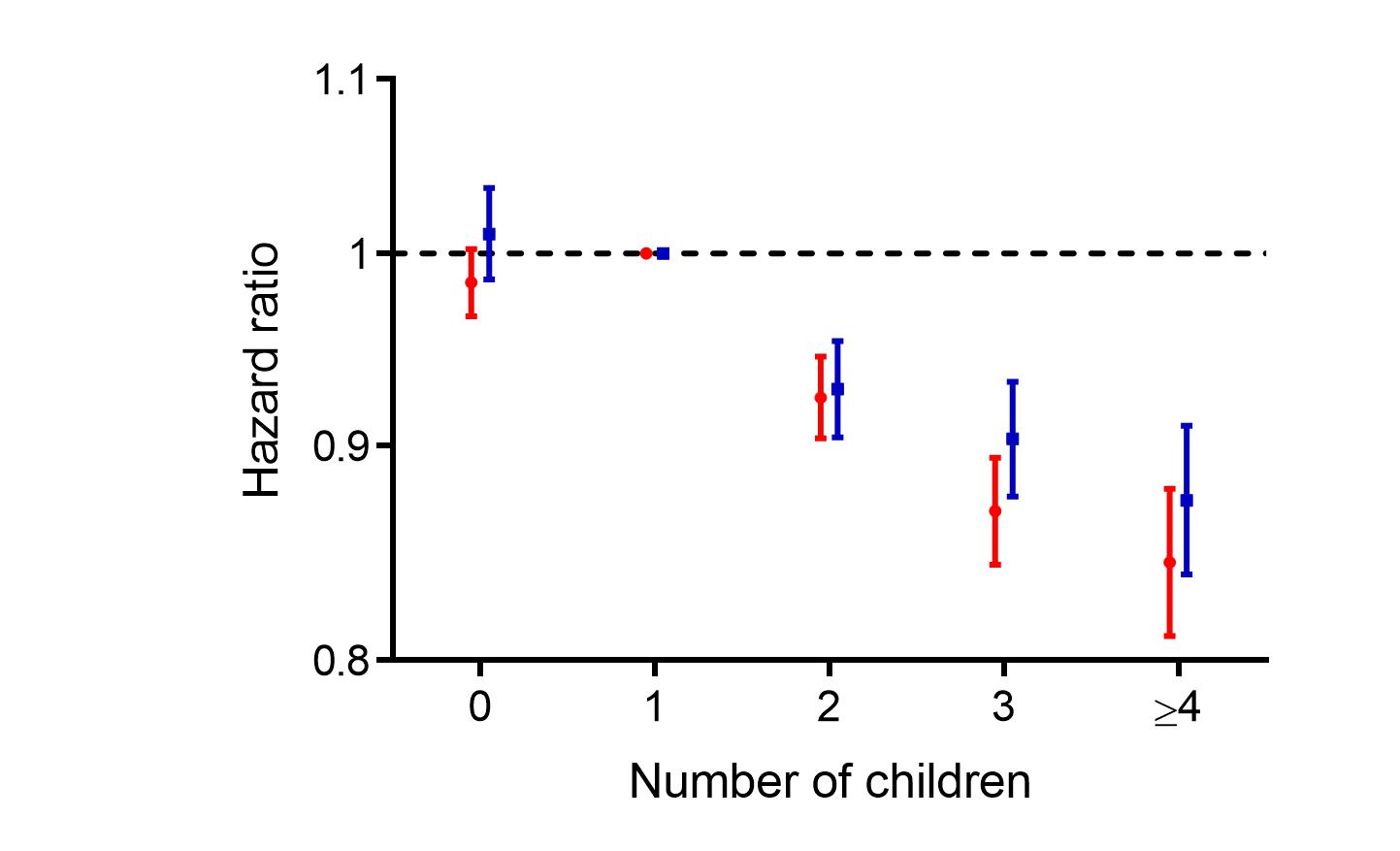


b)

a)

**Supplementary Figure 2. Associations between number of children and dementia, by dementia subtype, in a cohort of individuals ≥40 years old in the period 1994-2017 in Denmark.** Hazard ratios with 95% confidence intervals compare the risks of a) vascular dementia, b) Alzheimer’s disease, and c) other/unspecified dementia among women (red) and men (blue) with different numbers of children. All hazard ratios are adjusted for birth year (5-year intervals), cardiovascular disease, stroke, hypertension, chronic kidney disease and diabetes; age was the underlying time scale in the Cox model.


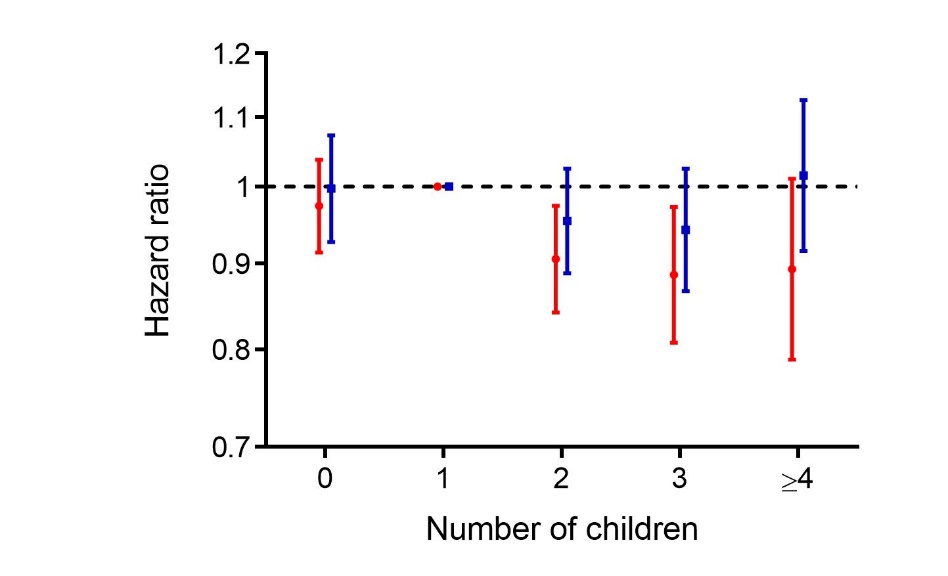

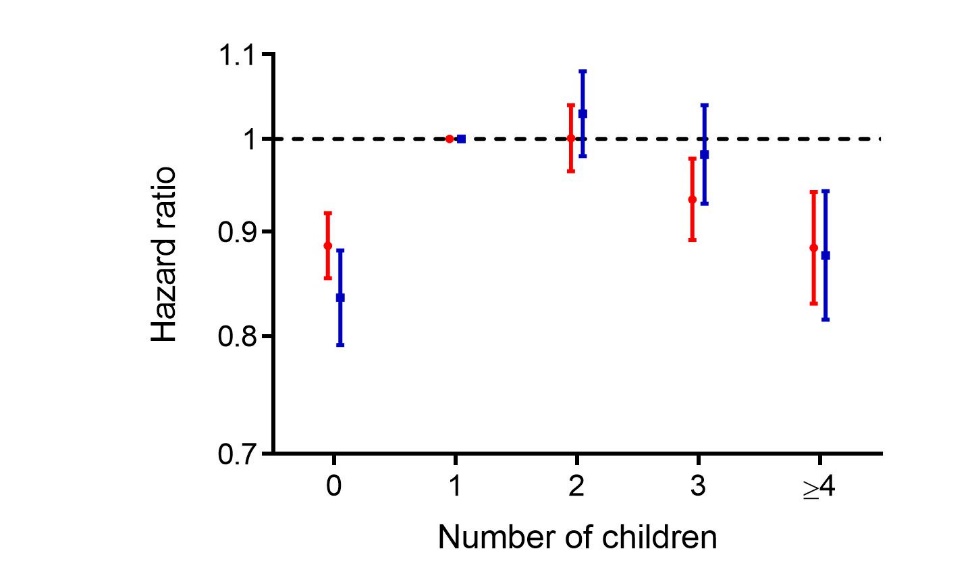

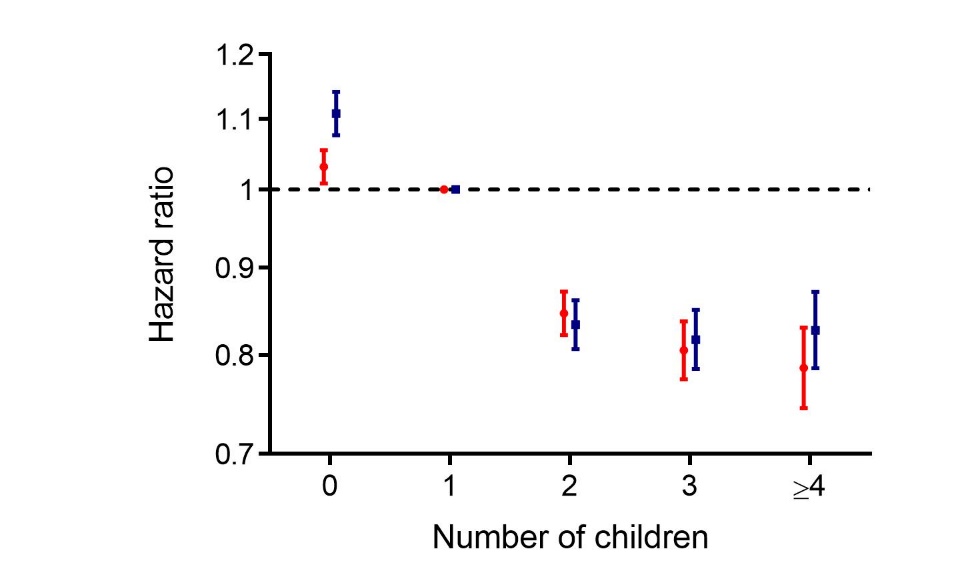


b)

a)

c))

**Supplementary Figure 3. Associations between number of children and dementia, by dementia subtype and timing of dementia onset, in a cohort of individuals ≥40 years old in the period 1994-2017 in Denmark.** Hazard ratios with 95% confidence intervals compare the risks of a) early-onset vascular dementia, b) late-onset vascular dementia, c) early-onset Alzheimer’s disease, d) late-onset Alzheimer’s disease, e) early-onset other/unspecified dementia, and f) late-onset other/unspecified dementia among women (red) and men (blue) with different numbers of children. All hazard ratios are adjusted for birth year (5-year intervals), cardiovascular disease, stroke, hypertension, chronic kidney disease and diabetes; age was the underlying time scale in the Cox model.


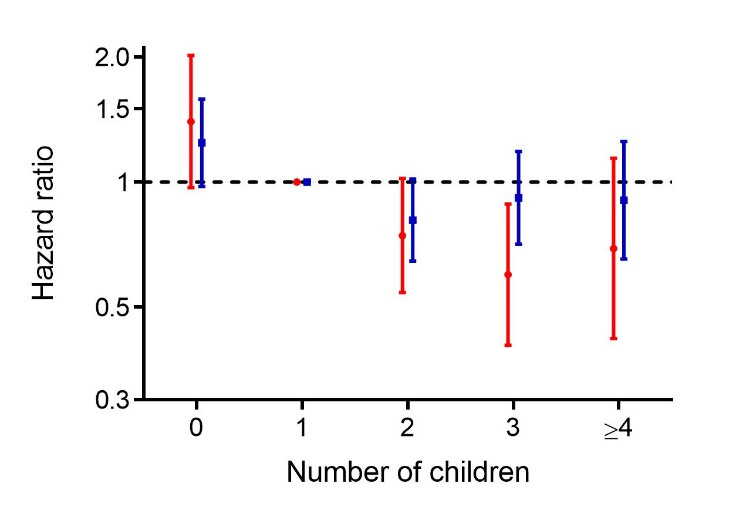

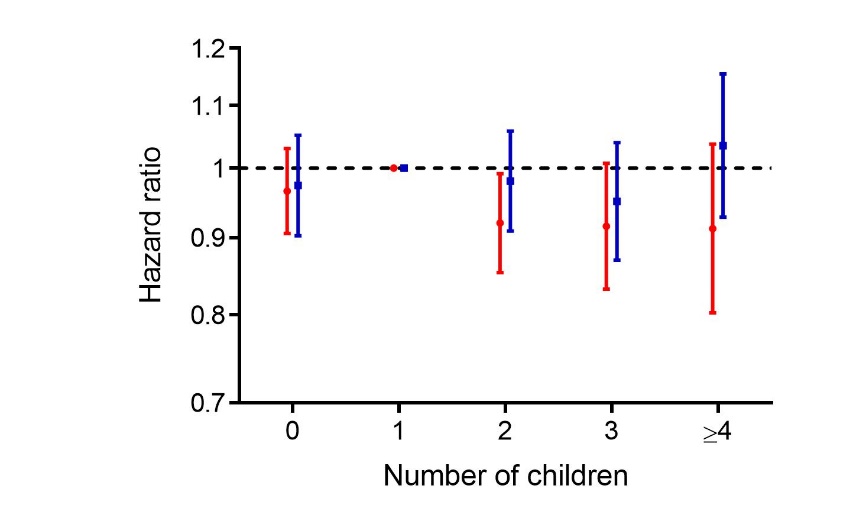

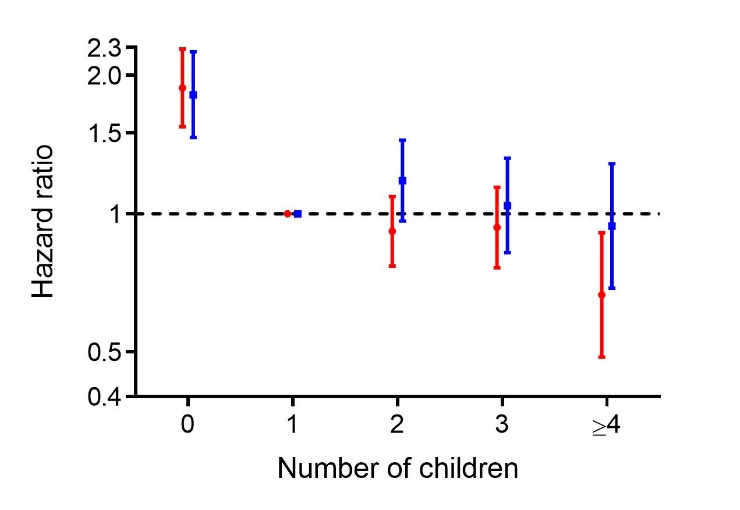

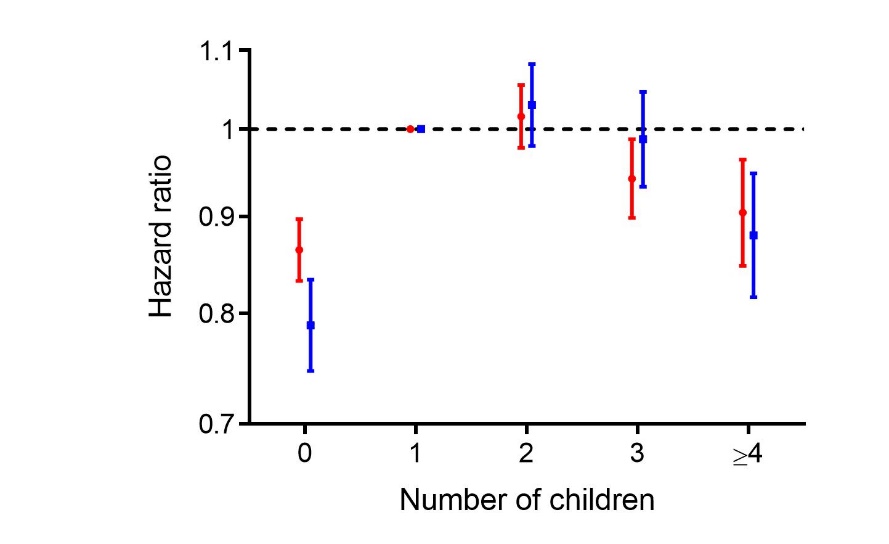

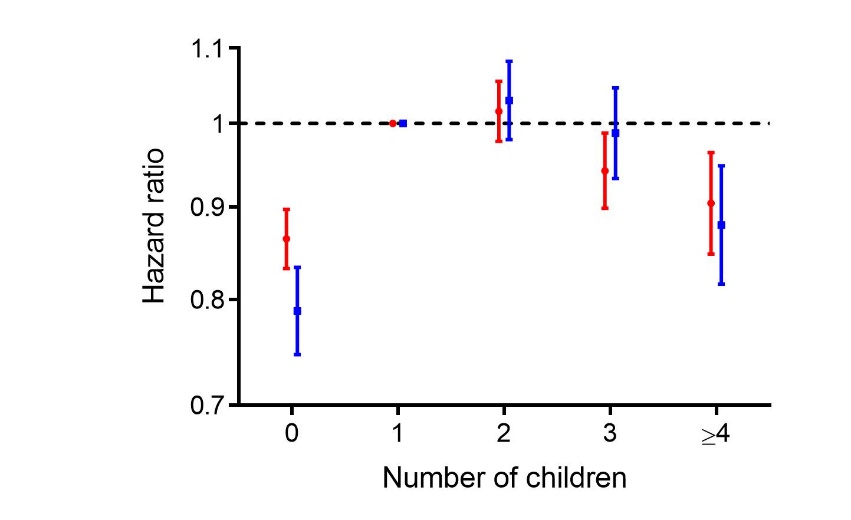

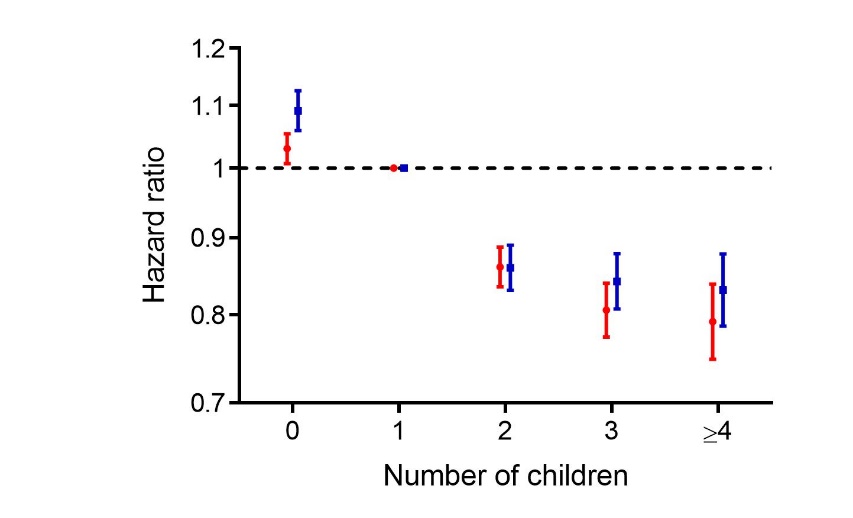


f)

e)

d)

a)

b)

c)

**Supplementary Figure 4. Associations between age at first birth and overall dementia, by timing of dementia onset, in a cohort of individuals ≥40 years old with ≥1 childbirths in the period 1994-2017 in Denmark.** Hazard ratios with 95% confidence intervals compare the risks of a) early-onset dementia (onset <65 years) and b) late-onset dementia (onset ≥65 years) among women (red) and men (blue) with different ages at first childbirth. All hazard ratios are adjusted for birth year (5-year intervals), cardiovascular disease, stroke, hypertension, chronic kidney disease and diabetes; age was the underlying time scale in the Cox model.


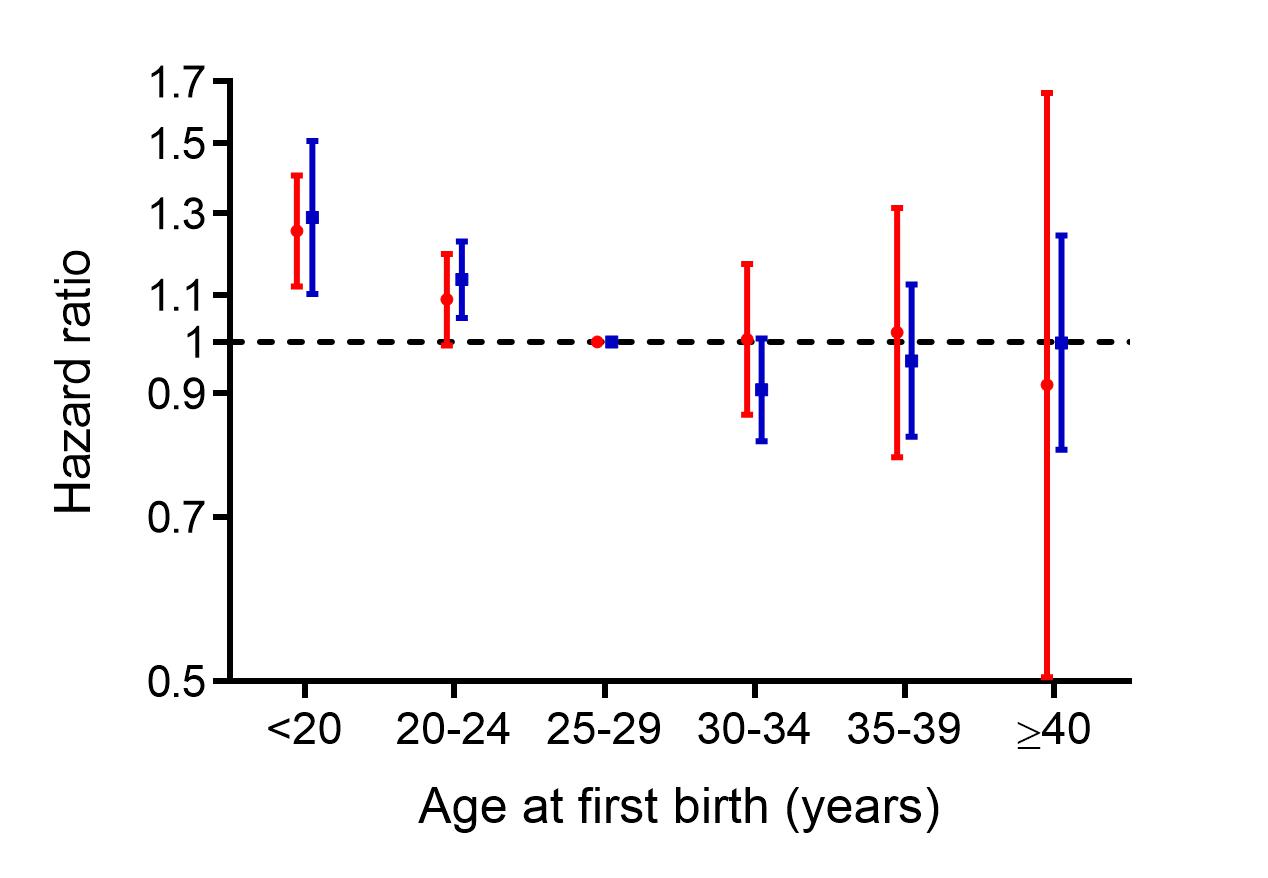

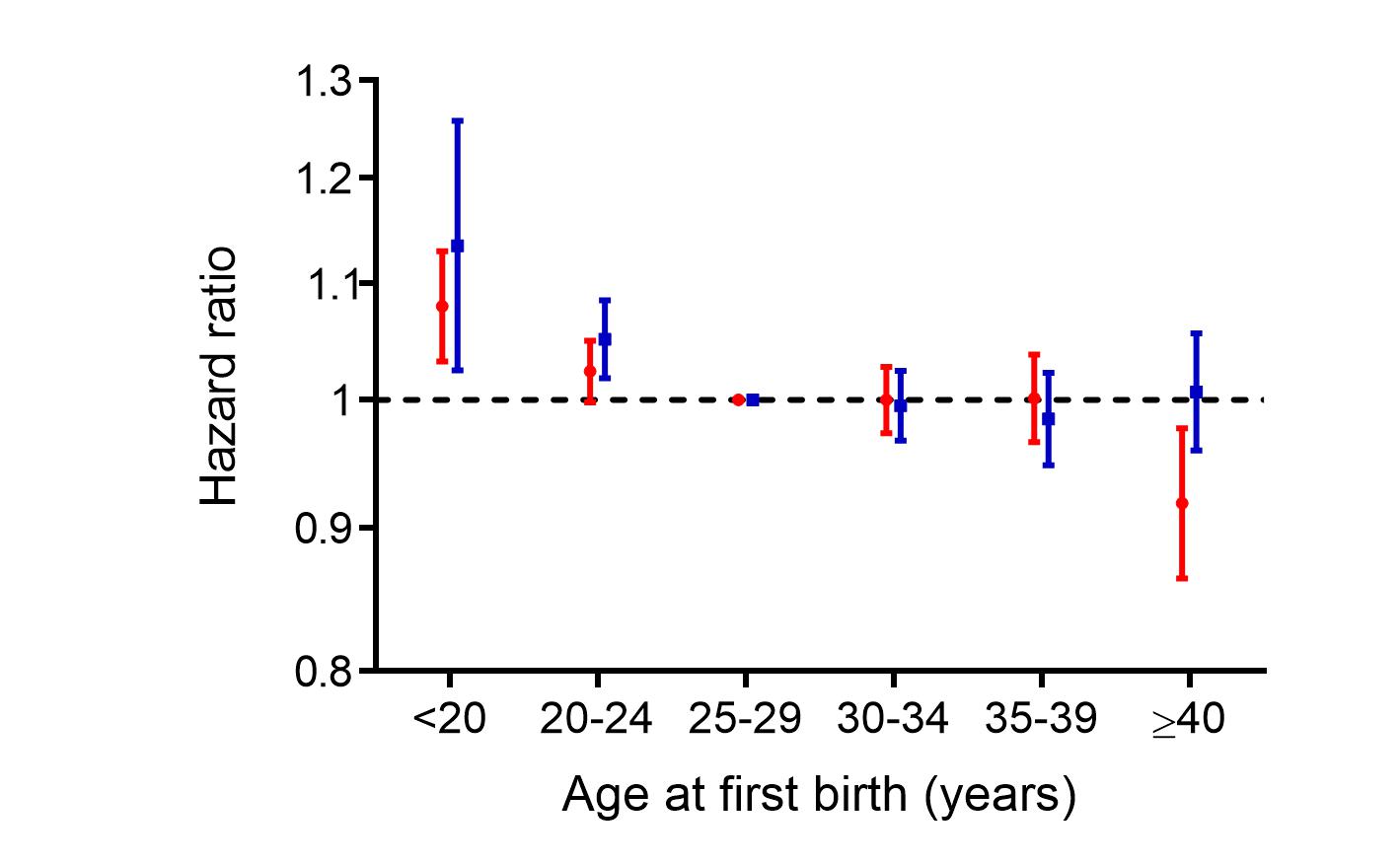


a)

b)

**Supplementary Figure 5. Associations between age at first birth and overall dementia, by dementia subtype, in a cohort of individuals ≥40 years old with ≥1 childbirths in the period 1994-2017 in Denmark.** Hazard ratios with 95% confidence intervals compare the risks of a) vascular dementia, b) Alzheimer’s disease, and c) other/unspecified dementia among women (red) and men (blue) with different ages at first childbirth. All hazard ratios are adjusted for birth year (5-year intervals), cardiovascular disease, stroke, hypertension, chronic kidney disease and diabetes; age was the underlying time scale in the Cox model.


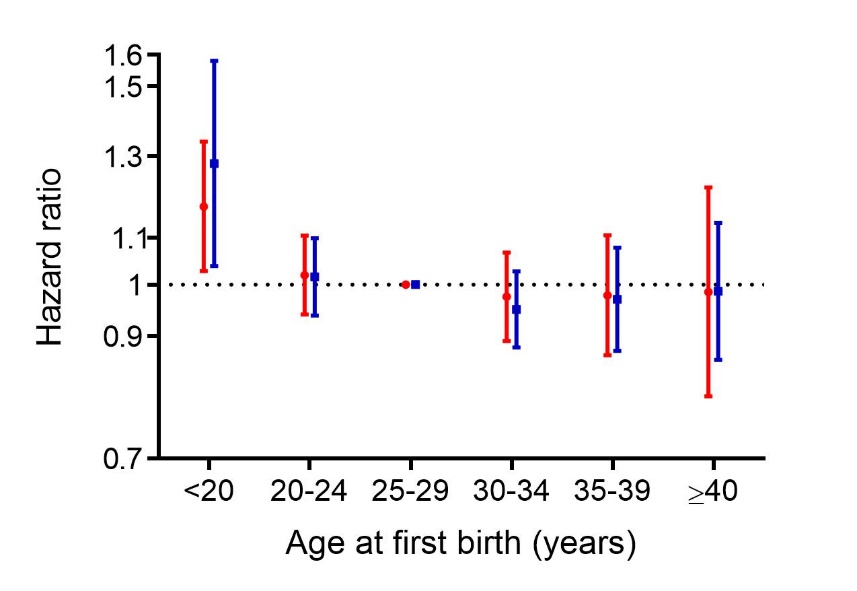

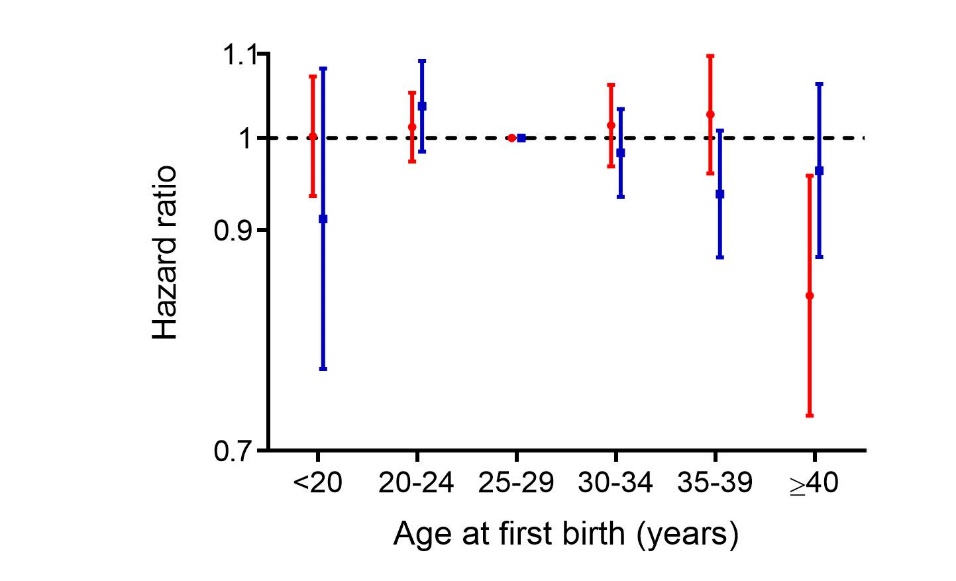

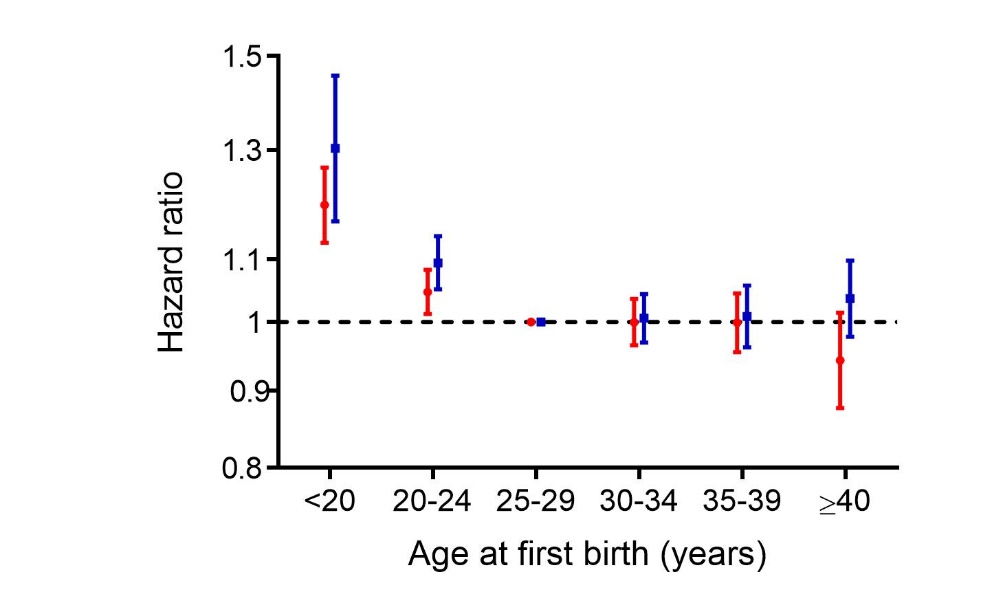


b)

a)

c)

**Supplementary Figure 6. Associations between age at first birth and overall dementia, by dementia subtype and timing of dementia onset, in a cohort of individuals ≥40 years old with ≥1 childbirths in the period 1994-2017 in Denmark.** Hazard ratios with 95% confidence intervals compare the risks of a) early-onset vascular dementia, b) late-onset vascular dementia, c) early-onset Alzheimer’s disease, d) late-onset Alzheimer’s disease, e) early-onset other/unspecified dementia, and f) late-onset other/unspecified dementia among women (red) and men (blue) with different ages at first childbirth. All hazard ratios are adjusted for birth year (5-year intervals), cardiovascular disease, stroke, hypertension, chronic kidney disease and diabetes; age was the underlying time scale in the Cox model.


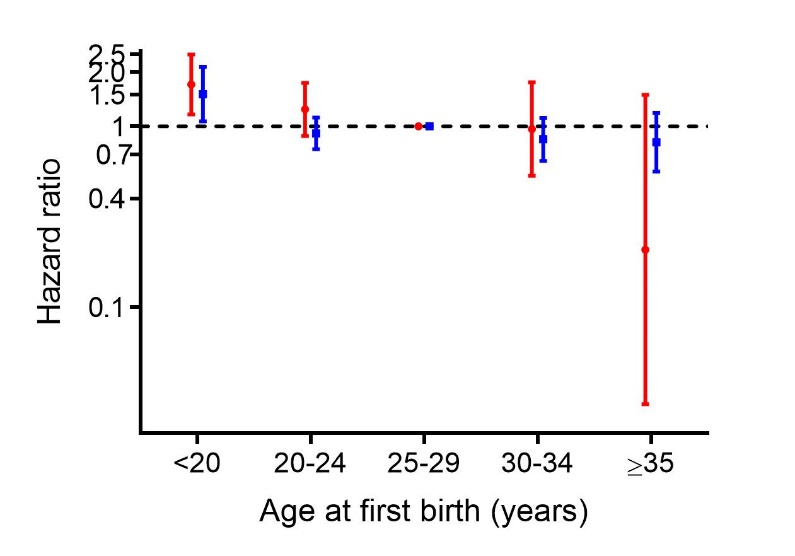

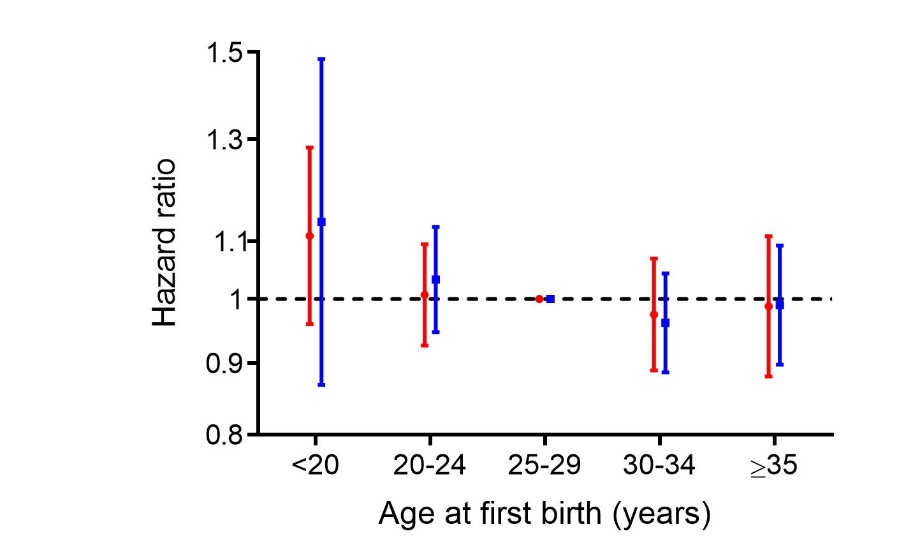

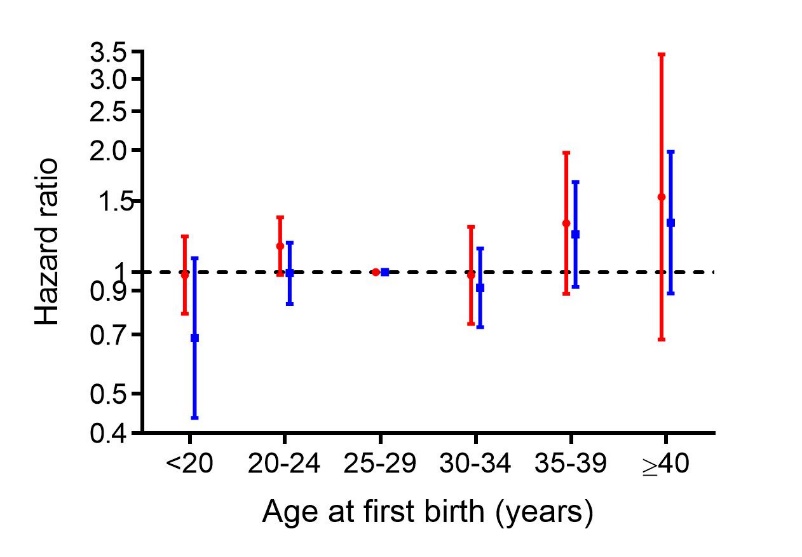

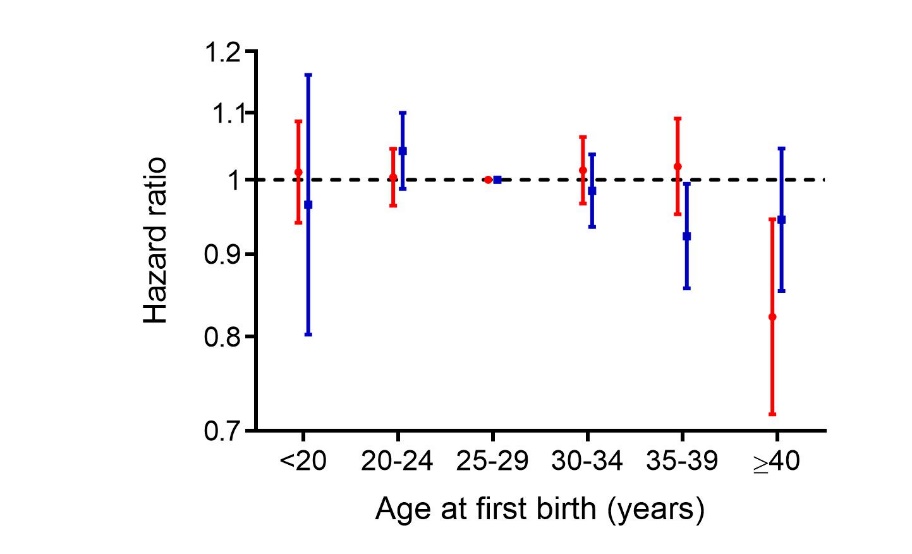

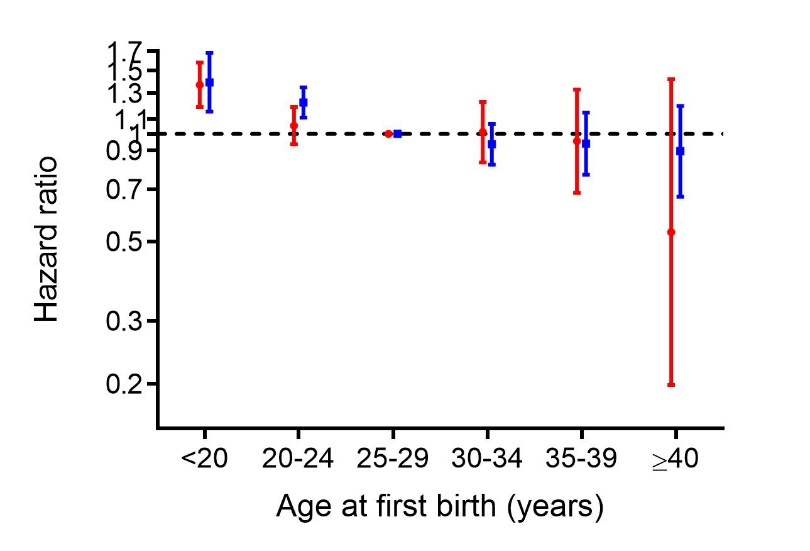

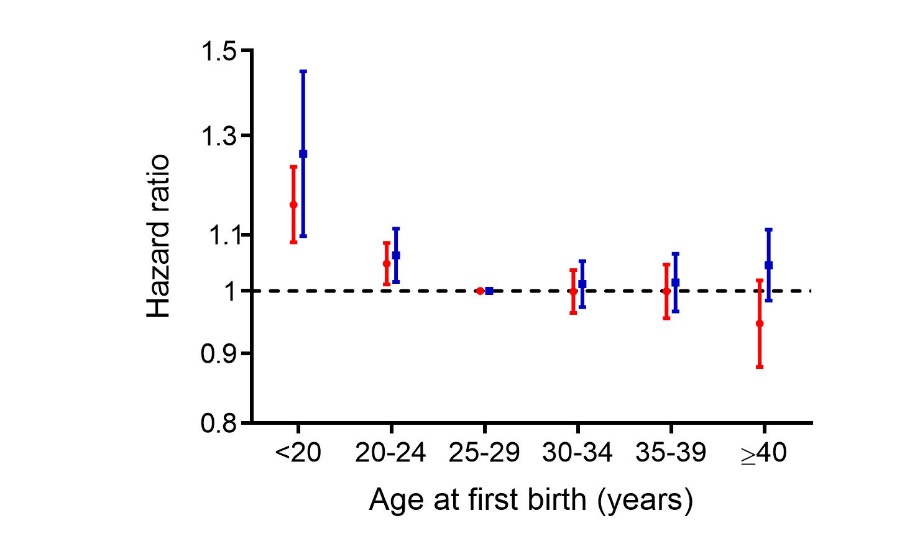


f)

e)

d)

c)

b)

a)
